# Transcriptomic and Metabolic Responses to a Live-Attenuated *Francisella tularensis* Vaccine

**DOI:** 10.3390/vaccines8030412

**Published:** 2020-07-24

**Authors:** Johannes B. Goll, Shuzhao Li, James L. Edwards, Steven E. Bosinger, Travis L. Jensen, Yating Wang, William F. Hooper, Casey E. Gelber, Katherine L. Sanders, Evan J. Anderson, Nadine Rouphael, Muktha S. Natrajan, Robert A. Johnson, Patrick Sanz, Daniel Hoft, Mark J. Mulligan

**Affiliations:** 1 The Emmes Company, Rockville, MD 20850, USA; jgoll@emmes.com (J.B.G.); tjensen@emmes.com (T.L.J.); willfhooper@gmail.com (W.F.H.); cgelber@emmes.com (C.E.G.); 2 Departments of Medicine, Emory University School of Medicine, Atlanta, GA 30322, USA; shuzhao.li@jax.org (S.L.); yating.wang@emory.edu (Y.W.); 3 Department of Chemistry, Saint Louis University, St Louis, MO 63103, USA; jim.edwards@slu.edu (J.L.E.); katherine.sanders@slu.edu (K.L.S.); 4 Yerkes National Primate Research Center, Secret Path, Atlanta, GA 30329, USA; steven.bosinger@emory.edu; 5 Emory Vaccine Center, Emory University, Atlanta, GA 30322, USA; nroupha@emory.edu (N.R.); muktha.natrajan@emory.edu (M.S.N.); 6 Department of Pathology and Laboratory Medicine, Emory University School of Medicine, Decatur, GA 30030, USA; 7 Division of Infectious Diseases, Department of Medicine, Emory University School of Medicine, Atlanta, GA 30322, USA; evanderson@emory.edu; 8 Department of Pediatrics, Emory University School of Medicine and Children’s Healthcare of Atlanta, Atlanta, GA, 30322, USA; 9 Biomedical Advanced Research and Development Authority, U. S. Department of Health and Human Services, Washington, DC 20201, USA; Robert.Johnson@hhs.gov; 10 Division of Microbiology and Infectious Diseases, National Institute of Allergy and Infectious Diseases, National Institutes of Health, Rockville, MD 20892, USA; patrick.sanz@nih.gov; 11 Division of Infectious Diseases, Allergy and Immunology, Saint Louis University Health Sciences Center, St. Louis, MO 63104, USA; Daniel.hoft@health.slu.edu; 12 Division of Infectious Diseases and Immunology, Department of Medicine, and New York University (NYU) Langone Vaccine Center, NYU School of Medicine, New York, NY 10016, USA

**Keywords:** tularemia vaccine, *Francisella tularenis* vaccine, DVC-LVS, *Francisella tularensis*, human immune response, RNA-Seq, metabolomics, LC–MS, innate immune signaling, TLR, TNF, NF-κB, NOD-like receptor, interferon α/β signaling, suppression of immune response

## Abstract

The immune response to live-attenuated *Francisella tularensis* vaccine and its host evasion mechanisms are incompletely understood. Using RNA-Seq and LC–MS on samples collected pre-vaccination and at days 1, 2, 7, and 14 post-vaccination, we identified differentially expressed genes in PBMCs, metabolites in serum, enriched pathways, and metabolites that correlated with T cell and B cell responses, or gene expression modules. While an early activation of interferon α/β signaling was observed, several innate immune signaling pathways including TLR, TNF, NF-κB, and NOD-like receptor signaling and key inflammatory cytokines such as Il-1α, Il-1β, and TNF typically activated following infection were suppressed. The NF-κB pathway was the most impacted and the likely route of attack. Plasma cells, immunoglobulin, and B cell signatures were evident by day 7. MHC I antigen presentation was more actively up-regulated first followed by MHC II which coincided with the emergence of humoral immune signatures. Metabolomics analysis showed that glycolysis and TCA cycle-related metabolites were perturbed including a decline in pyruvate. Correlation networks that provide hypotheses on the interplay between changes in innate immune, T cell, and B cell gene expression signatures and metabolites are provided. Results demonstrate the utility of transcriptomics and metabolomics for better understanding molecular mechanisms of vaccine response and potential host–pathogen interactions.

## 1. Introduction

*Francisella tularensis* is a highly pathogenic, Gram-negative bacterium and is listed as a category A bioterrorism agent (Dennis et al., 2001). We previously conducted a Phase 2 trial of two vaccines against *F. tularensis*, DVC-LVS and USAMRIID-LVS, which were based on two different lots of the same live vaccine strain. The clinical trial confirmed that both vaccines were safe and resulted in high rates of seroconversion (≥4-fold rise in tularemia-specific microagglutination titer at day 28 or 56) [1]. The molecular mechanisms underlying these seroconversions are not well understood. In addition, *F. tularensis* pathogenesis is enhanced by the bacterium’s ability to suppress host innate immune responses [2]. The mechanisms used by the bacterium to achieve this suppression are actively being researched.

The application of high-throughput technologies to assess cell-wide changes in gene expression, protein abundance, small molecules, and lipids opens a new opportunity to better understand the mechanisms of vaccines and human immune responses [3,4,5,6,7,8]. For example, transcriptomics studies to assess changes in gene expression in PBMCs or individual cell types revealed detailed information on immune signaling pathway activation following vaccination against H5N1 influenza when given with AS03 vaccine adjuvant and identified early markers that correlated with later protection [7,8]. Metabolomics measures global metabolite profiles, reflecting both cellular metabolism and systemic metabolite signals [9,10] and similarly promises to reveal biomarkers that correlate with vaccine protection or other phenotypes of interest. Although fewer metabolomics studies of vaccine responses have been conducted in comparison with transcriptomics, some metabolomics studies have been carried out. One such herpes zoster vaccine study [11] combined metabolomics and transcriptomics analyses and revealed that metabolic phenotypes greatly influenced changes in immune responses detected by transcriptomics and cellular immunology assays. An Ebola metabolomics vaccine study is in progress [12]. An integrative-omics analysis using ferrets infected with human pandemic H1N1 influenza demonstrated a correlation between the abundance of pro-inflammatory lipids and severe tracheal lesions [13]. Other studies showed that metabolites (e.g., phosphatidylserine, sphingosine-1-phosphate, resolvins and leukotrienes) direct immune-related signaling [14,15,16,17,18,19]. Despite recognition that metabolites are critical to the differentiation and function of immune cells, our understanding of the specific role of metabolites in the systemic immune responses remains limited [20].

Our goal in this study was to combine metabolic and transcriptomic profiling to reveal deeper insights into biological mechanisms through which tularemia live strain vaccination generated cellular and humoral immune responses. As a first step toward this goal, we previously assessed antibody responses, cell-mediated immune responses, and PBMC gene expression responses based on microarrays following vaccination with the two tularemia vaccines [1,21].

In this current study, we profiled changes in PBMC gene expression using RNA-Seq and in blood metabolites using LC–MS in samples obtained from ten subjects enrolled in our earlier clinical trial [1] who received the DVC-LVS live bacterial vaccine (the newer vaccine lot generated using current good manufacturing practices (cGMPs)). In both experiments, blood from day 0 (pre-vaccination) and from days 1, 2, 7, and 14 post-vaccination was sampled and processed. We also performed proteomics and lipidomics experiments on the same cohort and time points, which are described in referenced materials [22,23] and are cross-referenced in the discussion section.

Comparisons between pre- and post-vaccination samples revealed several transcriptomics and metabolic features that changed following vaccination. While certain innate immune signaling pathways were up-regulated, several pro-inflammatory pathways and associated cytokines were strongly suppressed on the gene expression level. Despite this attenuation of the innate immune response, gene expression signatures related to humoral immune responses emerged later during the time course. Furthermore, our analysis revealed that metabolic signatures related to energy metabolism, in particular, glycolysis and TCA cycle, were perturbed following vaccination. Finally, we identified metabolite biomarkers that best predicted later adaptive immune responses and correlated with transcriptomics responses.

Together, these results provide additional insights into molecular pathways and metabolites involved in the responses to DVC-LVS live tularemia vaccination and potential interactions between attenuated *F. tularemia* and its host.

## 2. Materials and Methods

### 2.1. Clinical Study

The parent clinical trial (NCT01150695) was designed as a double-blind, randomized study [1]. Subjects were randomly assigned to one of two vaccine groups. One group was vaccinated with a single, undiluted dose of the *F. tularensis* live vaccine strain (LVS) produced by DynPort Vaccine Company, Frederick, USA (DVC-LVS). The other group was vaccinated with a single, undiluted dose of the older stock-piled United States Army Medical Research Institute of Infectious Diseases vaccine (USAMRIID-LVS). Both vaccines were administered in the ulnar aspect of the volar surface (palm side) of the forearm, midway between the wrist and the elbow, via scarification (multiple puncture technique). In a prior substudy, blood samples were taken from 42 healthy male and female subjects aged 18 to 45 years old to assess transcriptomic responses using Affymetrix GeneChips [21]. In this study, we assessed transcriptomic and metabolomic signatures in 50 samples using RNA-Seq and LC–MS/MS, respectively, from 10 subjects in the DVC-LVS vaccination group only. Blood samples were collected at day 0 (pre-vaccination) and days 1, 2, 7, and 14 post-vaccination. The DVC-LVS vaccine recipients were selected for this study as the DVC-LVS vaccine was generated from the latest lot of tularemia vaccine manufactured in accordance with current good manufacturing practices (CGMPs).

### 2.2. Transcriptomics

#### 2.2.1. RNA-Seq Experiments

PBMCs were harvested before (day 0) and after DVC-LVS vaccination (days 1, 2, 7, and 14), placed in TRIzol RNA extraction buffer, and frozen at −80 °C. Aliquots from the same samples were independently processed by two different laboratories using 250 ng of total RNA as input.

Experiment 1 (Saint Louis University): Libraries were generated using the Illumina (San Diego, CA, USA) TruSeq v2 RNA library kits. Ribosomal RNA was removed by poly-A selection using Oligo-dT beads (mRNA Direct kit, Life Technologies). mRNA was reverse transcribed to yield cDNA using SuperScript III RT enzyme (Life Technologies, Carlsbad, CA, USA) and random hexamers. A second-strand reaction was performed to yield ds-cDNA. cDNA was blunt ended, had an A base added to the 3′ ends, and then had Illumina sequencing adapters ligated to the ends. Ligated fragments were then amplified for 14 cycles using primers incorporating unique index tags. Fragments were sequenced on an Illumina HiSeq-3000 using single reads extending 101 bases. All samples were processed in a single pool and sequenced over 5 lanes.

Experiment 2 (Emory University): Libraries were prepared using the Illumina (Illumina Inc. San Diego, CA, USA) TruSeq mRNA stranded kit as per the manufacturer’s instructions. In total, 250 ng of total RNA was used for library preparation. The TruSeq method (high-throughput protocol) employs two rounds of poly-A-based mRNA enrichment using oligo-dT magnetic beads followed by mRNA fragmentation (120–200 bp) using cations at high temperature. First- and second-strand cDNA synthesis was performed followed by end repair of the blunt cDNA ends. One single A base was added at the 3′ end of the cDNA followed by ligation of a unique barcode adapter for each sample. The adapter-ligated libraries were then enriched using PCR amplification. The libraries were normalized, pooled, and clustered on the Illumina cBot HiSeq3000/4000 flow cell. The clustered flow cell was then sequenced on the Illumina HiSeq3000 system employing a paired-end 151 cycle run (read length of 151 nt). All samples were processed in a single pool and sequenced over 5 lanes.

#### 2.2.2. RNA-Seq Data Processing and Analysis

The latest version of the human reference genome (GRCh38), gene models, and associated gene annotation information were obtained from the ENSEMBL database (Version 84, March 2016) [24]. The genomic reference was built by merging all human chromosomes except X and Y chromosomes to avoid gender-specific effects. Reads were mapped against the human reference genome using the STAR splice-aware read aligner (Version 2.5.2a) [25]. Gene expression quantification was carried out using the featureCounts function as implemented in the Subread software (Version 1.5.0-p2) [26]. TMM normalization was executed as implemented in *edgeR* [27]. Genes with expression levels below 8 CPM for all 50 samples were considered to be lowly expressed and were excluded from the differential gene analysis. To identify genes that were significantly differentially expressed (DE) from baseline for each post-vaccination day (days 1, 2, 7, 14), a negative binomial model was fit to read counts using the implementation provided by the *edgeR* software [27]. Each model included fixed effects for subject to account for paired samples and study visit day (baseline or post-vaccination day). For each gene, the statistical significance of the study visit day effect was evaluated using a likelihood ratio test. To control for testing multiple genes, the false-discovery rate (FDR) based on the Benjamini-Hochberg procedure as implemented in the p.adjust R function was applied for each model [28]. Genes with a fold change from baseline ≥ 1.5 and FDR-adjusted *p*-value < 0.05 were considered DE genes. Pathway enrichment analysis to identify significantly enriched KEGG [29], *MSigDB* [30], and Blood Transcription Modules (BTMs) [31] functional modules was carried out using the *goseq* R package [32] correcting for gene length bias and multiple testing (FDR-adjusted *p*-value < 0.1). See Supplemental Text for additional details.

#### 2.2.3. RNA-Seq Data Availability

Fragment counts are provided in Data File S1 and via the GEO database (accession GSE149809).

### 2.3. Metabolomics

#### 2.3.1. Amino Acid Sample and Data Processing

Plasma samples were precipitated with acetonitrile and spiked with deuterated amino acids (derived from algae, Cambridge Isotope Laboratories Inc., Tewksbury, PA, USA) and deuterated glutamate. Targets were 19 amino acids (cysteine was excluded due to notorious poor sensitivity in mass spectrometry). Multiple reaction monitoring was used to analyze for the targeted amino acids. Aspartate and threonine were below the detection limit and were designated N/D for “not detected”. Of the 17 remaining amino acids, 14 had correlating isotopic internal standards from the algae. The remaining 3 amino acids were quantified against their closest neighboring isotopic standard. The stable isotope amino acids were used to account for differences in sample preparation. The average ratio for each isotope amino acid was used as a multiplication factor for the signal intensities for each of the endogenous amino acids. Because the internal standards are set and the signal intensities are ratiometric, the endogenous metabolite micromolar concentrations were ascertained by the ratios of each amino acid.

#### 2.3.2. Organic Acid Sample Experiment

Plasma samples were precipitated with acetonitrile and spiked with deuterated succinate and deuterated methyl malonic acid. Targeted analytes included lactate, pyruvate, succinate, methylmalonate, phosphoenolpyruvate, malate, citrate and glucose-6-phosphate. Signal intensities from targeted analytes were normalized to the deuterated methylmalonate internal standard. Targeted organic acid quantification results for each sample were recorded as LC–MS peak intensity ratios (targeted analyte 1–8/d-methylmalonate peak intensity ratio).

#### 2.3.3. High-Resolution LC–MS Metabolomics Experiment

Plasma samples were processed using liquid chromatography (HILIC and C18 chromatography columns) coupled to a Thermo Q Exactive HF quadrupole-Orbitrap mass spectrometer (mass-to-charge *m*/*z* ratio range from 85 to 1275 Daltons, 120,000 resolution) via ESI. For each plasma sample, three technical replicates were run. Untargeted metabolite abundance was measured in peak MS intensity. The C18 experiment was run in negative ion mode while HILIC was run in positive ion mode. An in-house MS/MS metabolite database and xMSannotator was used to annotate features [33]. The xMSannotator software assigned different confidence levels of annotation based on matches to the HMDB database [34] and peak patterns within the data. Untargeted metabolites were labeled by mass-to-charge ratio (*m*/*z*) and retention time (rt) and by tentative chemical annotations if the xMSannotator confidence score was ≥2.

#### 2.3.4. Metabolomics Analysis

##### Filtering and Normalization of Metabolomics Data

Intensity data were log_2_ transformed. For the untargeted experiment, the median coefficient of variation (CV) per *m*/*z* feature based on its three technical replicates across samples was obtained. A feature needed to have three non-missing technical replicate observations for CV values to be calculated. Features with a median CV that was below the lower (25%) quartile of the overall median CV distribution (ignoring missing CVs) were recorded for inclusion in the differential metabolite analysis. Technical replicates per sample were combined using the mean log_2_ intensity. Systematic differences between samples were corrected for using median normalization (See Supplemental Text for additional details).

##### Missing Value Imputation

Missing observations in the untargeted experiments were imputed using the k-nearest neighbors algorithm implemented in the impute R package (Version 1.44.0) [35]. Only metabolites with at least 40/50 (80%) non-missing observations were used as input for imputation and downstream analysis. The number of neighbors to be used as part of the imputation step was set to 8. The maximum percentage of metabolites with missing observations per sample was set to 80%. For targeted experiments, missing observations were not imputed and were excluded from the analysis (See Supplemental Text for additional details and assessments of missingness).

##### Identification of Differentially Abundant Metabolites

Metabolites that significantly differed in their response from baseline were identified by using a two-sided permutation paired t-test comparing baseline (day 0) to post-vaccination (day X) metabolite signals (H_0_:μ(day_X_–day_0_) = 0, H_1_:μ(day_X_–day_0_) ≠ 0; on the log_2_ scale). Untargeted *m*/*z* features were considered differentially abundant (DA) if their FDR-adjusted *p*-value was <0.4 and their fold change from pre-vaccination was ≥1.2 in either direction. For targeted experiments, an individual *p*-value cut-off of <0.05 was applied to determine statistical significance.

##### Identification of Metabolomics Responses that Best Predict Adaptive Immune Response

Regularized linear regression models were fit to determine C18 and HILIC metabolite *log*_2_-fold change responses that best predicted peak percent activated CD4 cells, peak percent activated CD8 cells, and peak microagglutination titer using the *glmnet* R package (Version 2.0-13) [36]. Leave-one-out cross-validation was used to determine the optimum regularization parameters *α* and *λ* that minimized the model mean squared error. The input metabolite set included all median-normalized metabolite *log*_2_-fold changes for a given day. Prior to modeling, metabolite *log*_2_-fold changes were standardized. Models were considered to fit the data well if they achieved an R^2^ of at least 0.7.

##### Integration of Metabolomics and Transcriptomics Results

To identify correlated transcriptomics and metabolomics responses, we applied similar methods as described in Gardinassi et al. [37]. Both omics datasets were first collapsed into functional modules/pathways. For transcriptomics, this was accomplished using Blood Transcription Modules (BTMs) calculating the mean *log*_2_-fold change of gene expression per BTM and clustering BTMs by Pearson correlation distance. Multiscale bootstrapping as implemented in the pvclust R package [38] was used to identify robust BTM clusters (1000 permutations, unbiased bootstrap probability ≥0.95 and maximum distance of 0.5). Metabolites were filtered to retain metabolites with a minimum average fold change of >1.2. Filtered metabolites were then clustered based on their *log*_2_-fold change using Pearson correlation distance. Pearson correlation distance was scaled based on distance in retention time. Metabolite clusters were identified using multiscale bootstrap resampling using the same parameters as for BTMs. Next, partial least squares regression (PLS) analysis was carried out for each pairwise BTM and metabolite cluster pair, and the total *Q*2 was calculated for the first component using leave one out cross-validation. The input matrices for PLS included module *log*_2_-fold changes for each BTM cluster member and individual fold changes for each metabolite cluster member. The null distribution of *Q*2 for the first PLS component was determined using random resampling that maintained the same cluster sizes (10,000 random draws for each pair of cluster sizes). The *p*-value was calculated as the number of random draws that had a *Q*2 of equal or greater value than the observed *Q*2, divided by the total number of random draws. PLS models with an FDR-adjusted *p*-value < 0.05 were summarized.

##### Data Availability

Analysis datasets for the targeted metabolomics experiment are provided in Data Files S2–S4.

### 2.4. Ethical Statement

Subjects gave their consent for inclusion before they enrolled in the parent clinical trial [1]. The protocol and consent form were reviewed by the US Food and Drug Administration, and approved and monitored by the clinical sites’ institutional review boards.

## 3. Results

In this study, high-dimensional measurements of gene expression and metabolites were obtained over five time points from 10 subjects who received the DVC-LVS vaccine [1]. RNA-Seq results were independently obtained at two laboratories by using RNA-Seq on aliquots derived from the same 50 collected samples using slightly different experimental protocols (see Materials and Methods). As RNA-Seq results between experiments were very similar, we decided to present results for the experiment that did not have any outliers (Experiment 1) and show results for the less complete set (Experiment 2) as part of the supplement as additional evidence (see Supplemental Text). We first report vaccine-induced PBMC gene expression signals and then describe metabolite changes post-vaccination. Finally, the associations between metabolites and adaptive immune responses and the associations between metabolites and gene expression modules are explored to generate mechanistic hypotheses.

### 3.1. PBMC Gene Expression Following Tularemia Vaccination Showed Increases over Time in Magnitude and Complexity with the Majority of Genes Being Down-Regulated

**Figure 1:**
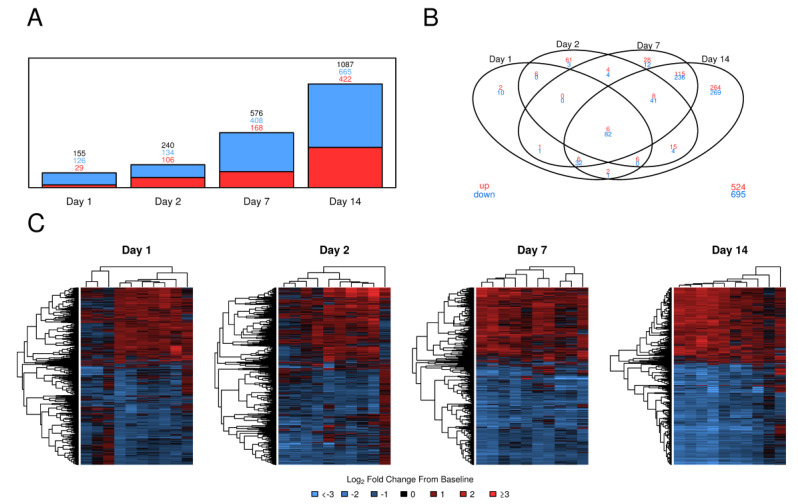
PBMC gene expression changes over time following tularemia live-attenuated vaccination. (**A**): Barplot summarizing DE genes over time by up/down-regulation. In red: up-regulated; in blue: down-regulated; in black: up- or down-regulated. (**B**): Venn diagram of DE genes over time. (**C**): Heatmaps of *log*_2_-fold change from pre-vaccination. Genes were hierarchically clustered using uncentered Pearson correlation of *log*_2_-fold changes in combination with the complete linkage algorithm. Gene order may differ between heatmaps.

To assess changes in the PBMC transcriptomes of 10 subjects over time, we identified differentially expressed (DE) genes for each post-vaccination day and visualized responses using barplots, Venn diagrams, and heatmaps (Figure 1, Table S1). An increase in vaccine effect both in terms of the number of DE genes as well as in fold change and consistency across subjects was observed across days 1, 2, 7, and 14 post-vaccination. By day 7, most subjects showed consistent results in terms of up/down-regulation (Figure 1C) and most DE genes at day 7 continued to be DE at day 14 (Figure 1B). The majority of DE genes were down-regulated. Among all time points, 88 DE genes were shared—of which, 82 (93%) were down-regulated relative to pre-vaccination. Very similar results were observed for the second RNA-Seq experiment (Text Figure S6).

### 3.2. Tularemia Vaccine Activated PBMC Gene Expression Patterns Related to Interferon Signaling But Repressed Other Cytokine and Innate Immune Signaling Pathways Involved in Inflammation

**Figure 2:**
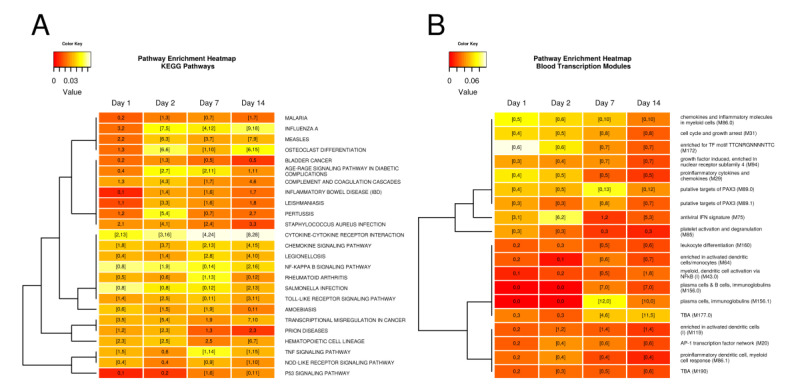
Heatmap of pathways that were enriched in PBMC DE genes. (**A**): KEGG pathways. (**B**): Blood Transcription Modules. Pathways significantly enriched in at least two conditions are shown. Cells are color coded by the Jaccard Similarity Index which measures the agreement between DE genes and pathways. Lighter colors indicated stronger agreement. Cells contain the number of DE genes in a pathway with DE gene numbers in brackets indicating significantly enriched sets. The first number indicates up-regulated DE genes, the second number represents down-regulated DE genes. Pathways were clustered based on the Jaccard distance between their binary enrichment pattern.

To functionally characterize DE responses in the context of known pathways, we carried out pathway enrichment analysis (Figure 2, Table S2, Text Figure S7). IFN-*α*/*β* signaling was enriched at day 2 (Source Reactome, Table S2) with all 14 DE genes in this pathway being up-regulated. However, several innate immune signaling pathways were enriched in down-regulated DE gene responses at days 1, 2, 7, and 14; in particular, the cytokine–cytokine receptor interaction pathway showed the highest degree of enrichment and an increase over time both in enrichment and in the number of down-regulated DE genes (>78% of DE genes) (Figure 2A). Enriched innate immune response signaling pathways included Toll-like receptor (TLR) signaling (recognizes microbial pathogens and triggers innate immune responses), NOD-like receptor signaling (detects intracellular pathogens and triggers innate immune response), TNF-signaling (triggers intracellular signaling related to apoptosis/cell survival as well as inflammation and immunity), and NF-κB signaling (regulates genes involved in immunity, inflammation, and cell survival) (Figure 2A). There were generally an increasing number of DE genes per pathway indicating an increase in complexity of these innate responses over time. However, enrichment of these pathways was dominated by DE genes that were down-regulated following live-attenuated *F. tularensis* vaccine.

**Figure 3:**
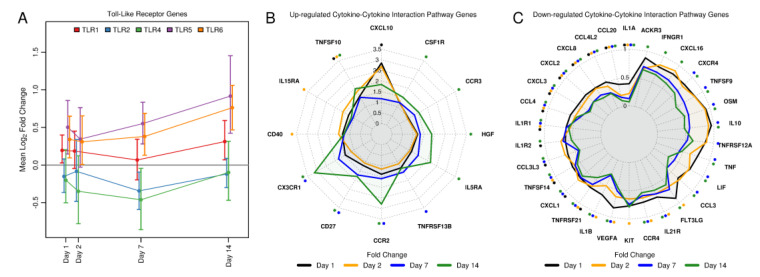
PBMC gene expression responses for Toll-like receptor signaling and cytokine–cytokine receptor interaction pathway genes. (**A**): Mean fold change and associated 95% confidence interval of toll-like receptor genes over time. (**B**): Radar plot of up-regulated DE genes in the cytokine–cytokine receptor interaction pathway. (**C**): Radar plot of down-regulated DE genes in the cytokine–cytokine receptor interaction pathway. Asterisks indicate statistically significant changes that are color coded by day.

Next, we assessed toll-like receptor (TLR) signaling and cytokine-signaling over time in more detail using time trend plots for TLR-encoding genes, radar plots of up and down-regulated DE genes in the cytokine–cytokine receptor interaction pathway (Figure 3, Text Figure S8), and color-coded pathway maps (Text Figures S19–S26 and S56–S62). TLR pathway maps showed a day 2 up-regulation of genes encoding for TLR proteins (*TLR1*, *TLR6*, and *TLR5*), which represent receptors that are known to recognize bacteria. In addition, gene expression for the interferon-inducible transcription factor *STAT1* was significantly up-regulated and expression of the gene that encodes for the IP-10 cytokine was increased (Text Figures S56 and S60). Expression for *TLR6* and *TLR5* strongly increased at day 7 reaching peak response at day 14 (Figure 3A). In contrast, *TLR2* and *TLR4* showed decreased responses relative to pre-vaccination, in particular at day 7. An important pathway downstream of TLR signaling and activation of cytokine expression and inflammation is the NF-κB signaling pathway. The NF-κB signaling pathway map and enrichment results demonstrated that many genes in this pathway were increasingly repressed over time (Figure 2, Text Figures S33–S40).

The strongest increase among DE genes encoding for cytokine signaling was observed for the IP-10-encoding *CXCL10* gene, with a day 1 DE response that was increased >2.5-fold relative to pre-vaccination (Figure 3B). Chemokine receptor-encoding genes *CCR2* and *CX3CR1* were significantly increased from pre-vaccination at day 7 and particularly at day 14. In addition, the tumor necrosis factor super family member 10 (*TNFSF10*) gene was significantly up-regulated at day 1, 2, and 14 with highest responses at day 14. However, the majority of DE genes in the cytokine–cytokine receptor interaction pathway were down-regulated and key pro-inflammatory cytokines were most substantially suppressed with a statistically significant >2-fold reduction in expression relative to pre-vaccination for days 1, 2, 7, and 14 for *IL-1α* and days 7 and 14 for *IL-1β* and *TNFα* (Figure 3C). All three of these cytokines are linked to the NF-κB signaling pathway. Similar results were observed for the second RNA-Seq experiment (Text Figure S8).

The PPARγ receptor is known to play a role in resolving inflammation. Increasing pathway enrichment results were found for several published PPARγ-related Immunologic Signature Gene Sets including GSE25123 WT vs. PPARG KO MACROPHAGE UP (up-regulated gene expression signals in wild type macrophages versus macrophage-specific PPARγ knockouts in mice) [39], which had 13 overlapping DE genes at day 1 and 51 overlapping DE genes with GSE25123 CTRL vs. ROSIGLITAZONE STIM PPARG KO MACROPHAGE_UP [39] at day 14 (Table S2).

Pathway enrichment profiles for Blood Transcription Modules (BTMs) showed activation of several inflammation and cytokine-related BTMs across all four post-vaccination days including chemokines and inflammatory molecules in myeloid cells (M86.0), proinflammatory cytokines and chemokines (M29), and proinflammatory dendritic cell, myeloid cell response (M86.1), with the strongest enrichment signals at days 1 or 2 (Figure 2B, Text Figure S7B). While these three BTMs were enriched in down-regulated DE genes, the antiviral IFN signature (M75) BTM was activated, supporting our findings based on KEGG and Reactome enrichment results that interferon-related signaling was activated.

### 3.3. Tularemia Vaccine Induced Gene Expression Signals Related to the Adaptive Immune Response at Day 7 and 14

Plasma cell, immunoglobulin, and B cell-related BTMs (plasma cells, immunoglobulins (M156.1), and plasma cells and B cells, immunoglobulins (M156.0)) were enriched in up-regulated PBMC DE gene responses at days 7 and 14, with stronger enrichment signals at day 7 (Figure 2B, Text Figure S7B). Our previously published results showed that an adaptive immune response measured using a tularemia-specific microagglutination titer was readily detectable by day 14, the first post-vaccination time point assayed for *F. tularensis*-specific antibody [1], and by day 7 on the gene expression level for the DVC-LVS group [21]. The BTM enrichment results from this study confirmed that adaptive immune response signal-related gene expression signals were detected in PBMCs as early as day 7.

### 3.4. Changes in Plasma Amino and Organic Acids within 2 Days after Tularemia Vaccination

**Figure 4:**
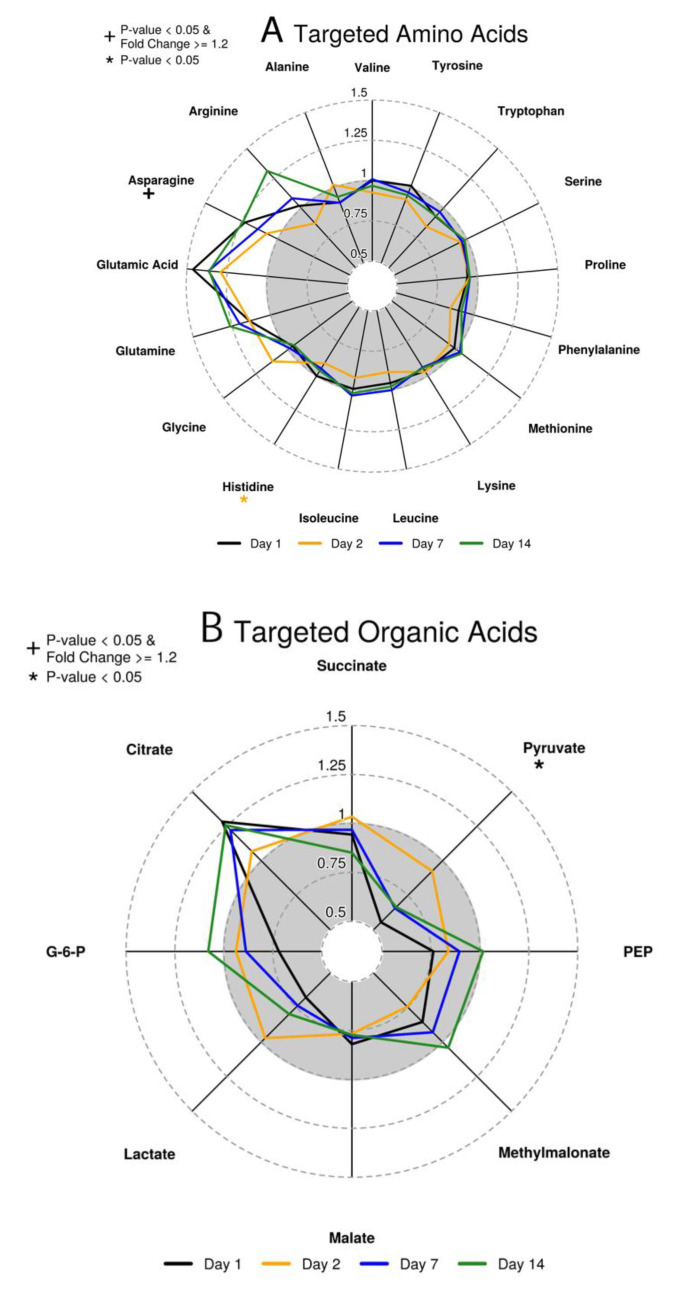
Radar plots summarizing changes in targeted amino and organic acids. (**A**): amino acids; (**B**): organic acids. Each ray represents one targeted metabolite. Each line presents the mean fold change compared to pre-vaccination for a particular post-vaccination day. Asterisks indicate statistically significant up-regulation (*p*-value < 0.05), while a plus symbol represents statistical significance (*p*-value < 0.05) and a fold change of ≥ 1.2. The grey area marks fold changes below 1 indicating that metabolite abundance was lower than pre-vaccination for the respective metabolite and post-vaccination time point.

Using targeted mass spectrometry assays, we measured the molar concentration of 17 amino acids and 8 organic acids. Radar plots that summarize fold changes for targeted plasma amino acids and organic acids are shown in Figure 4. Among organic acids, pyruvate was identified as a differentially abundant (DA) metabolite at day 1 with a 44% decreased mean micromolar concentration compared to baseline. The amino acid asparagine was DA with a 23% increased mean micromolar concentration compared to baseline at day 1. In addition, histidine levels were statistically significantly decreased by 9% at day 2.

### 3.5. Untargeted Metabolomics Revealed Changes in Energy Metabolism and Nucleotide Metabolism

**Figure 5:**
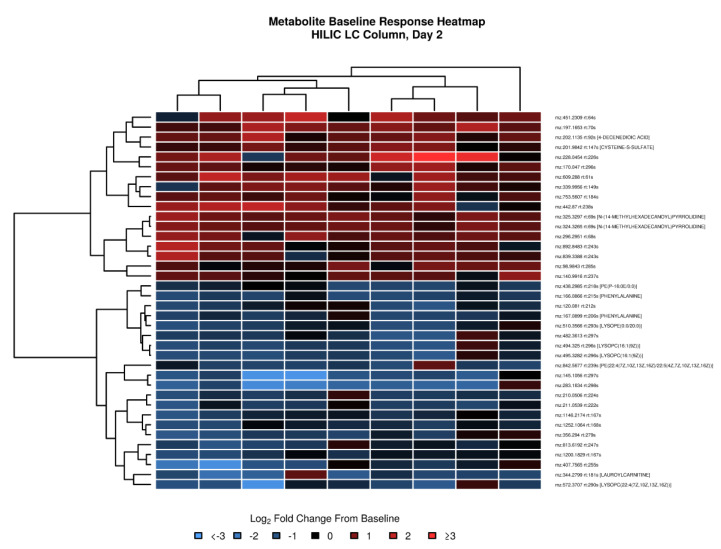
Heatmap of day 2 *log*_2_-fold changes from pre-vaccination for metabolites identified using the HILIC column. Rows represent DA metabolites, and columns represent samples. In red: increased signal compared to pre-vaccination; in blue: decreased signal compared to pre-vaccination. Dendrograms were obtained using complete linkage clustering of uncentered pairwise Pearson correlation distances between *log*_2_-fold changes. Metabolites are labeled by mass-to-charge ratio (*m*/*z*) and retention time (rt). Tentative chemical annotations are provided for metabolites with an annotation confidence score ≥ 2. For metabolites with multiple annotations, the first annotation is shown. “D-” or “L-”, “trans-” or “cis-” were removed from the annotations as the applied LC–MS method cannot distinguish these cases.

High-resolution untargeted metabolomics was performed on plasma samples using a dual column setup of LC–MS. Overall, 3741 and 6562 unique metabolite *m*/*z* features were identified for C18 and HILIC, respectively, for which at least 80% of samples had recorded intensity values. Of these, 1208 and 2107 for C18 and HILIC, respectively, passed the lower quartile CV criterion for technical replicates to be included in differential analysis. For the HILIC column, when compared to day 0 pre-vaccination levels, 38 significant (FDR-adjusted *p*-value < 0.4 and fold change of ≥1.2) metabolite *m*/*z* features were found at day 2 (Table S3). No DA features were identified for the other post-vaccination days. Heatmap analysis confirmed that subjects tended to have similar responses for DA metabolites that were either concordantly increased or decreased from pre-vaccination (Figure 5). For the C18 column, 21 DA metabolite features were identified at day 1 and 49 were identified at day 14—7 of which overlapped between day 1 and 14. All DA metabolites and heatmaps are provided in Table S3 and Text Figures S187–S189, respectively.

Given that annotation on untargeted metabolomics is challenging, we utilized mummichog software [40] to predict KEGG metabolic pathway enrichment directly from the untargeted data. The top pathways enriched in day 2 HILIC DA metabolites are shown in Table S4. The top enriched pathway was 2-oxocarboxylic acid metabolism, which links glycolysis and the TCA cycle, corroborating the significant change of pyruvate in the targeted analysis. While arginine and glutamic acid did not meet our criterion for a statistically significant response in our targeted analysis, they did show decreased and increased mean responses at day 2, respectively (Figure 4). These changes are consistent with enriched metabolic pathways in Table S4 involving arginine and 2-oxocarboxylic acid metabolism. In addition, Purine metabolism was enriched. Purine metabolism is part of nucleotide metabolism, and is possibly related to the proliferation of immune cells induced by vaccination.

### 3.6. Exploring Metabolite Predictors of Antibody and T Cell Responses

**Figure 6:**
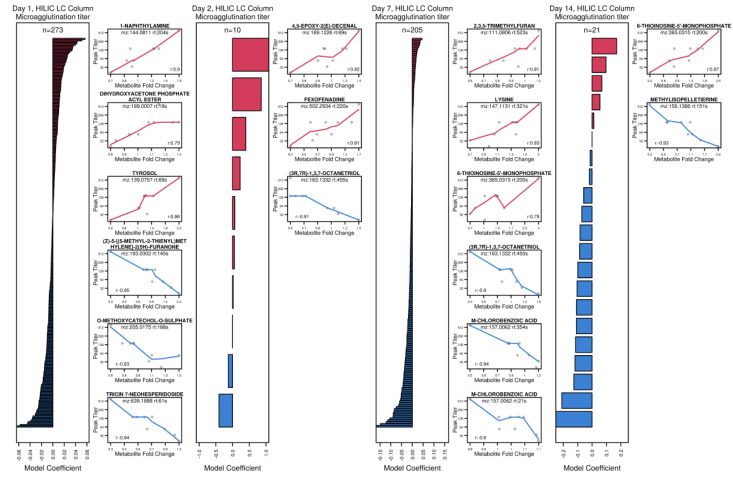
Metabolite changes on days 1, 2, 7 and 14 that best predicted peak microagglutination titer. Barplots and scatterplots summarizing regularized linear regression results for metabolites that best predict peak microagglutination titer for each time points. Barplots visualize linear regression coefficients. Scatterplots for the top and bottom three metabolite features based on the linear regression coefficients with tentative chemical annotations (confidence score ≥ 2) are shown for each day. For metabolites with multiple annotations, the first annotation is shown. “D-” or “L-”, “trans-” or “cis-” were removed from the annotations, as the applied LC–MS method cannot distinguish these cases.

A critical question for the field of vaccinology is whether metabolite profiles can predict the downstream cellular and/or adaptive responses that are fundamental to immunological protection against pathogens. Here, we employed elastic net regularized linear regression, which was successfully applied to similar contexts [7,8], to test metabolites as predictors of antibody response (peak tularemia-specific microagglutination titer) or peak CD4+ and CD8+ T cell activation. The input set included *log*_2_-fold changes for all metabolites that were in the final analysis dataset (3741 C18 and 6562 for HILIC). Leave one out cross-validation was used to fine-tune the model based on the mean squared error. Both alpha and lambda were tuned and models that achieved an R-squared of ≥0.7 were considered well-performing models. Day 1–14 changes in HILIC-based metabolites predicted peak microagglutination titer. Overall, 273, 10, 205, and 21 metabolites were identified for days 1, 2, 7, 14, respectively (Table S5). Barplots that contrast the magnitude of regression coefficients of predictive metabolites and scatterplots for the top and bottom three ranking metabolites with metabolite annotations are provided in Figure 6. Day 2 changes in HILIC-based metabolites predicted CD4+ and CD8+ T cell activation with 250 and 32 predictive metabolites, respectively (Table S5, Text Figures S209 and S210). Day 2 changes in 25 C18-based metabolites predicted peak microagglutination titer (Table S6, Figure S206). Day 1 and 2 changes in C18-based metabolites predicted peak CD8+ T cell activation with 153 and 198 predictive metabolites, respectively (Table S5, Figure S207).

### 3.7. Significant Correlations between Genes and Metabolites and Association with Immune Responses

To investigate correlated events between plasma metabolomics and PBMC transcriptomics in recipients of tularemia vaccine, we adopted methods published in Gardinassi et al. [37]. Briefly, both omics datasets were first collapsed into functional modules/pathways using Blood Transcription Modules (BTMs) [31] for transcriptomics and hierarchical clustering based on distances scaled by retention times for metabolomics. Overall, 44 BTM, 53 HILIC metabolite, and 8 C18 metabolite clusters were identified. Partial least squares regression (PLS) analysis resulted in 110 significant cluster associations (Table S6). A selection of correlations between BTMs and metabolite variables based on the first component are summarized in Figure 7. Results for all significant networks are shown in Figures S211–S320.

**Figure 7:**
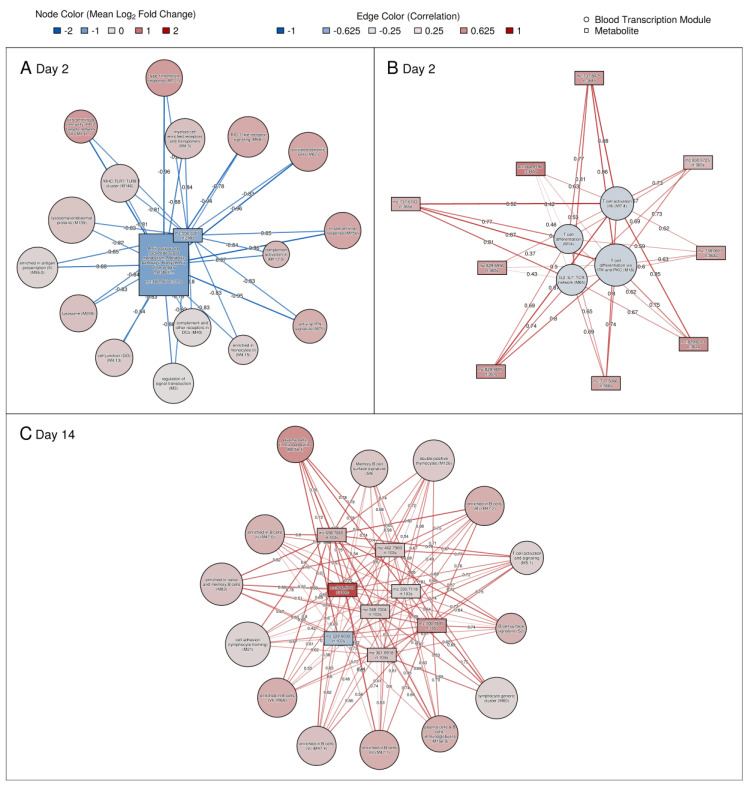
Blood Transcription Module gene expression changes that were correlated with metabolite changes. Node color gradient encodes fold change from pre-vaccination. In red: up-regulated compared to pre-vaccination; in blue: down-regulated compared to pre-vaccination. Edges represent correlation between Blood Transcription Modules and metabolite responses. Edge thickness and edge color is scaled with increasing correlation. Circles represent Blood Transcription Modules. Boxes are used for metabolites. Metabolites are labeled by mass-to-charge ratio (*m*/*z*) and retention time (rt). In addition, mappings to KEGG metabolic pathways are included as part of metabolite labels if a matching KEGG compound could be identified. For example, the metabolite peak with *m*/*z* = 180.087 and rt = 211s is a tentative match to protonated glucosamine by accurate mass and was linked to the Amino sugar and nucleotide sugar metabolism and Metabolic KEGG pathways (Table S6). (**A**): Day 2 IFN-γ signature; (**B**): Day 2 T cell signature; (**C**): Day 14: B cell signature. KEGG pathway information was added for metabolites with an annotation confidence score ≥ 2 based on HMDB mappings.

The day 2 network presented in Figure 7 shows that the up-regulation of several innate immune signaling-related BTM gene modules strongly negatively correlated with the down-regulation of two metabolites (Q^2^ = 0.76) (Figure 7A). The strongest up-regulated modules included type I interferon response (M127), viral sensing and immunity; IRF2 targets network (II) (M111.1), RIG-1 like receptor signaling (M68), and activated dendritic cells (M67). Both metabolites in this cluster were decreased from pre-vaccination. One (*m*/*z* 180.087) was matched to glucosamine while the other had no database match. Nine metabolites with intensities that were increased from pre-vaccination at day 2 were significantly positively associated with increases in T cell activation/differentiation-related BTM gene modules, and these metabolites were enriched in the steroid biosynthesis pathway (Q^2^ = 0.42) (Figure 7B). Although these T cell BTM gene modules had mean expression levels close to pre-vaccination at day 2, the association may shed light on potential mechanisms of T cell activation. Figure 7C summarizes associations between 8 primarily up-regulated metabolites and 14 B cell and plasma cell-related BTM gene modules for day 14.

## 4. Discussion

The effective component of the DVC-LVS vaccine is live-attenuated *F. tularensis*, a highly pathogenic bacterium whose strong virulence has been attributed to its ability to evade and alter the host immune response, and, in particular, to disrupt the host’s innate immune response including inflammatory response [2,41,42,43,44,45].

Our transcriptomics results showed that while the number of DE genes in PBMCs steadily increased over time, reaching peak numbers at day 14, the majority were down-regulated compared to pre-vaccination. This included key innate immune signaling signatures that are normally activated by the host in response to intracellular pathogens. In particular, we saw a higher degree of down-regulated DE genes involved in cytokine–cytokine receptor interaction and NF-κB signaling, which have been shown to be targeted and suppressed by *F. tularensis* to temper pro-inflammatory response [44,45]. The same was true for the TLR, TNF, and NOD-like receptor signaling pathways. This indicated that while the complexity of vaccine-induced PBMC gene expression signals developed over time, they represented not only true host responses to ward off the bacterium, but likely also host responses altered by the bacterium to evade it.

Toll-like receptors are critical to the initial innate immune response against bacteria such as *F. tularensis*. These membrane receptors, which are located on the outside and inside of innate immune cells, detect extracellular and intracellular pathogens by recognizing pathogen-associated molecular patterns (PAMPs) [46]. Recognition mediates pro-inflammatory response triggering the production of cytokines and activation of antigen-presenting cells and is instrumental for shaping later antigen-specific adaptive immune response. Inspection of TLR-signaling following *F. tularensis* vaccination revealed that the *TLR5* and *TLR6* genes that encode for receptors that recognize bacteria at the cell surface (bacterial flagellin and microbe-associated molecular patterns (MAMPs), respectively) were initially up-regulated starting at day 1 post-vaccination, with strongly increased levels by day 7 and reaching peak expression levels at day 14. In contrast, *TLR2* and *TLR4* gene expression levels either remained close to pre-vaccination levels or were down-regulated, as was observed for day 7. *TLR4* encodes for a receptor that is known to recognize lipopolysaccharide (LPS) present on Gram-negative bacteria such as *F. tularensis*. *F. tularensis* LPS has been shown to not strongly activate *TLR4,* potentially explaining the absence of this signal from our study [47,48]. *F. tularensis* triggers *TLR2* signaling via its Tul4 lipoprotein, resulting in the expression of pro-inflammatory cytokines [49]. *TLR2* has been shown to be essential to contain pulmonary *F. tularensis* infection in mice [50]. *F. tularensis* LVS triggered an increase in *TLR2* expression in murine macrophages, with peak responses during the first 12 h [51]. One possible explanation for the lack of up-regulation of *TLR2* gene expression in this study could be that the peak *TLR2* signal may have occurred prior to our first sampling time point 24h post-vaccination and was subsequently repressed by the pathogen. Another study of *F. tularensis* LVS demonstrated that *TLR2* as well as *TLR6* were important for TLR-mediated activation of the NF-κB pathway and inflammatory response in dendritic cells [52]. Despite being strongly up-regulated in our study, neither *TLR6* nor *TLR5* expression (nor any other changes in TLR expression) activated NF-κB pathway signaling and associated pro-inflammatory cytokine expression in our clinical cohort. The activation of this pathway requires the phosphorylation, ubiquitination, and degradation of IκB proteins that inhibit the entry of the NF-κB transcription factor into the nucleus by masking nuclear signaling motifs [53]. The phosphorylation, ubiquitination, and nuclear translocation steps have been a prime target of pathogens to avoid degradation of IκB and activation of the transcription factor [54,55]. NF-κB pathway analysis showed that expression of genes that encode for inhibitory IκB proteins was slightly decreased at days 1 and 2, and statistical significantly decreased at days 7 and 14 (>1.8-fold), indicating that NF-κB would have been activated relative to pre-vaccination considering constant levels of IκB phosphorylation, ubiquitination, and degradation (Text Figures S33–S40). We thus hypothesized that *F. tularensis* suppressed this pathway not by interfering with IκB but by interfering with the nuclear translocation step of the NF-κB transcription factor, given the lack of expression of its target genes. Indeed, a study in murine macrophages showed that the FTT0831 protein of the *F. tularensis* SchuS4 strain was able to block nuclear translocation of the NF-κB p65 subunit [56]. Based on these results, it is likely that this *F. tularensis* live-attenuated vaccine shut down gene expression of key pro-inflammatory cytokines including IL-1α, IL-1β, and TNFα through FTT0831 protein-mediated blocking of the NF-κB pathway.

Interferon α/β signaling was not impacted, as was evident by a significant enrichment in up-regulated DE genes by day 2. In addition, gene expression for the IP-10-encoding *CXCL10* gene was strongly increased from pre-vaccination at day 1 and 2 (>2.5-fold). IP-10 is a cytokine involved in multiple pro-inflammatory immune system processes including the activation and chemoattraction of immune cells. Chemokine receptor-encoding gene *CCR2* was significantly up-regulated at day 7 and 14. The receptor, when bound by CCL2, mediates macrophage and monocyte chemotaxis and regulates T cell differentiation into pro-inflammatory T-helper 17 cells. A similar profile with an up-regulation at day 7 and 14 was seen for *CX3CR1,* which encodes for a receptor that mediates leukocyte migration. *TNFSF10*, which is known to regulate apoptosis, was up-regulated for all post-vaccination days, with significant responses at days 1, 2, and 14. This implies that these cytokines were either not targeted by *F. tularensis* defense mechanisms or were expressed in dominant PBMC cell populations that were not infected.

Peroxisome proliferator-activated receptor γ (PPARγ)-related gene sets were enriched in down-regulated DE genes, particularly at day 14. A PPARγ signal was also observed in our related proteomics study which showed an enrichment of PPARγ-related gene sets in proteomics responses at day 14 [22]. PPARγ are nuclear receptor proteins that act as transcription factors regulating gene expression related to cell differentiation, metabolism, tumorigenesis, and inflammatory response. They can be activated by fatty acids of the 5-hudroxyeicosatetraenoic acid (5-HETE) family [57]. Interestingly, lipidomic analyses of the same samples in our related study revealed increased metabolism (decreased abundance) of the pro-inflammatory 5-hudroxyeicosatetraenoic acid (5-HETE) lipid by day 7 post-vaccination, associated with a compensatory increase in dihydroxyeicosatetraenoic acid (DHET) lipid levels [23]. The pro-inflammatory function of 5-HETE is known to be regulated through conversion to its inactive and less active metabolites, DHET lipids, by the Cytochrome P450F family of proteins. Indeed, in our transcriptomics data, we saw that gene *CYP4F22* was statistically significantly up-regulated on day 2 post-vaccination (1.7-fold increase), day 7 post-vaccination (1.6-fold increase), and day 14 post-vaccination (1.8-fold increase) (Table S1). Several Cytochrome P450 family genes including *CYP1B1* and *CYP4V2* were also differentially expressed. These results support the conclusion that 5-HETE is induced early in the response to *F. tularensis* vaccination, followed by induction of Cytochrome P450 gene expression and subsequent conversion of 5-HETE into inactive DHET at day 7.

Immunoglobulin-related BTMs showed strongest enrichment signals at day 7, which continued through day 14, with all DE genes mapping to this BTM being up-regulated relative to pre-vaccination. These PBMC gene expression patterns indicated that a humoral immune response likely occurred at these times. These results are consistent with our published microarrays results for which we identified an up-regulation of an immunoglobulin-related gene expression signature starting at day 7 [50]. Antigen processing and presentation is essential for a successful humoral response. Our concurrent proteomics results for the same cohort of subjects indicated that several proteins involved in MHC I antigen processing and presentation had significantly increased protein levels at day 7 [22]. Gene expression signatures in this study showed that MHC II genes were slightly up-regulated at day 2 including genes encoding for the MHC II molecule with the MHC II molecule being DE and up-regulated by day 7 (Text Figures S166–S173). Several key MHC I genes involved in loading intracellular peptides onto MHC I molecules (*PA28*, *HSP70*, and *TAP1/2*) were strongly activated at day 2, while genes encoding for the MHC I molecule showed a slight increase at day 2 with levels returning to pre-vaccination by day 7. Together, these results suggest that the pathway geared toward presenting antigens from intracellular pathogen proteins via MHC I was activated first followed by the presentation of extracellular pathogen proteins via MHC II at the day 7 sampling time point at which the initial humoral response signals emerged.

The elevation of serum asparagine levels on day 1 in our targeted metabolomics analysis indicated either an increase in aspartate transaminase activity or a reduction in asparaginase. The formation of asparagine occurs exclusively through transamination of aspartic acid, and the primary degradation of asparagine occurs through asparaginase hydrolysis. Levels of asparagine in serum are generally >10-fold higher than aspartic acid which suggests the preference of maintaining asparagine as the nitrogen carrier in the blood. This study showed that while asparagine increased at day 1, aspartic acid levels did not significantly change. Previous work showed that external asparaginase and depletion of asparagine inhibited T cell activation [58]. As the depletion of asparagine reduced T cell immune responses, the elevation of serum asparagine identified in this vaccine study could be important for optimal vaccine-induced immune activation. To the best of our knowledge this is the first instance of elevated asparagine found in response to vaccination. Decreases in histidine levels observed on day 2 were small but statistically significant and possibly occurred due to the conversion of histidine to histamine through carboxylase activity. Glutamine and glutamate levels both trended upward after vaccination. Previous work indicated that glutaminase, which converts glutamine to glutamate, was highly expressed in lymphocytes and macrophages and increased upon inflammatory stimuli [59]. The role of glutamine and glutaminase is largely centered on interleukin production and optimizing rates of DNA, RNA, and protein synthesis by lymphocytes.

At day 1 post-vaccination the glycolytic intermediate pyruvate was decreased in plasma. Additionally, levels of the glycolytic metabolites glucose-6-phosphate, phosphoenolpyruvate, and lactate were lower following vaccination, with glucose-6-phosphate and phosphoenolpyruvate levels returning to pre-vaccination levels by day 14. Furthermore, citrate levels were elevated throughout the time course. Citrate is an important immunomodulator that can be converted to produce important mediators such as reactive oxygen species (ROS) and prostaglandins. In addition, citrate can be metabolized to itaconic acid, which has antimicrobial functions [60,61]. The increase in citrate may be a result of increased activity of the Krebs cycle relative to glycolysis or a result of increased activity of fatty acid metabolism. LPS, which are present on the outer membrane of Gram-negative bacteria, have been shown to shift metabolism from oxidative phosphorylation to aerobic glycolysis following TLR signaling and activation of mouse macrophages and dendritic cells to increase inflammatory response including cytokine production [62,63]. A study that assessed metabolic reprogramming in the context of *F. tularensis* showed that the *F. tularensis* capsule was capable of suppressing macrophage aerobic glycolysis following TLR signaling as was evident by a relative decrease in lactate [64]. Given that lactate levels were generally reduced relative to pre-vaccination and that key pro-inflammatory cytokines were down-regulated, we hypothesize that suppression of aerobic glycolysis by attenuated *F. tularensis* may have occurred in this cohort.

Pathway enrichment analysis of untargeted plasma metabolites demonstrated that the galactose metabolism, glycolysis/gluconeogenesis, and the 2-oxocarboxylic acid metabolism pathway, which connects glycolysis to the TCA cycle, were enriched in DA metabolites at day 2. In addition, several amino acid metabolism, nucleotide metabolism (purine metabolism) and translation-related pathways (aminoacyl-tRNA biosynthesis) were perturbed at day 2. Perturbation of these pathways based on changes in plasma metabolites may be linked to an increase in innate immune cell activity/signaling requiring additional energy for support of protein translation and protein biosynthesis.

Strong associations between blood transcriptomics and metabolomics were shown previously in the context of vaccination [11] and infection [14]. Here, we assessed the interplay between transcriptomics and metabolomics in the context of a tularemia vaccine. For example, change in two metabolites including glucosamine, an amino sugar, were negatively correlated with an increase in innate immune signaling/activation BTMs (Figure 7A). Of great interest are the metabolites that were associated with plasma and B cell BTM gene signatures as these gene signatures have predicted antibody responses in prior vaccine studies [4,65,66]. For the BTM that showed the strongest up-regulation and correlation with metabolites (Plasma cells, Immunoglobulins (M156.1)), we also observed a weak positive correlation (r_s_ = 0.4) between log day 14 tularemia-specific microagglutination titer and the BTM day 14 log_2_ mean fold change response. Of the eight metabolites that were associated, all but one had increased levels at day 14 compared to pre-vaccination. Overall, these data indicate that significant associations between blood metabolomics and transcriptomics can be identified after tularemia vaccination. Together, such results can provide insights into the interaction between metabolic and cellular signaling events during the immune response.

Limitations of this study include its relatively small sample size and the focus on measuring only host transcriptional response via poly-A-based mRNA enrichment, which prevented us from co-detecting *F. tularensis*-specific RNA (we screened the data but did not find *F. tularensis*-specific RNA). While it has been shown that transcriptomics using RNA-Seq on bulk PBMCs can recapitulate most dominant signals of individual cell populations [7], our results cannot be attributed to particular cell types within PBMCs. RNA-Seq results that were independently generated by two laboratories produced similar outcomes lending additional confidence to the results. Sampling metabolites from plasma and RNA from PBMCs may not directly assay responses in the same cellular compartments, and both could be affected by immunological and physiological events outside of the blood circulation. It is important to note that the metabolite changes described here are found in serum whereas most existing work examined intracellular metabolites. Metabolite changes found in serum may be the result of an influx of metabolites from the bloodstream to accommodate the needs of the affected cells. Therefore, an inverse relationship between the serum metabolites and the needs of the receiving cell may exist, rather than a mirroring of the serum metabolites to the intracellular metabolite changes. Recent studies suggest that these systems cooperate in numerous pathways [67,68]. While we report accurate mass and retention times, the coverage and quality of metabolite annotations for our untargeted experiments is limited: 20% of C18 and 25% of HILIC data were annotated with at least one known compound with a confidence score ≥ 2. Thus, although these data confirm the feasibility of using metabolites at early time points to predict the later immune responses, the lack of annotations indicates that further work is required to utilize this information more effectively.

Our study is among the early efforts to investigate the metabolomics of vaccination and, to our knowledge, the most comprehensive RNA-Seq study of a *F. tularensis* live-attenuated vaccine in humans to date. The largest published human transcriptomics *F. tularensis* live-attenuated vaccine study carried out by others profiled gene expression pre- and 18 h, 42 h (day 2), 192 h (day 7), and 336 h (day 14) post-vaccination with the older tularemia vaccine lot (USAMRIID-LVS) [69]. Notable differences in experimental design and statistical analysis compared to our study included vaccine lot (USAMRIID-LVS vs. DVC-LVS), first post-vaccination time point (18 h vs. day 1), sample size (5 vs. 10), assay (microarray vs. RNA-Seq, total RNA vs. mRNA), and determination of modulated genes (2-fold vs. 1.5-fold up/down regulation, no adjustment for multiple testing vs. FDR adjustment). Both studies showed peak up-regulation of the MHC-I antigen processing-related *TAP1* gene at Day 2. While an early up-regulation for *TLR1*, *TLR5*, and *TLR6* was seen in both studies, trends differed for later time points. The time trend of *TLR1* observed in our study was closely mirrored by that of the Fuller et al. study, while *TL5* and *TLR6* showed peak response at day 14 in our study only. In the Fuller et al. study, *TLR4* was most strongly up-regulated at 18 h and remained up-regulated at day 2. In contrast, in our study, *TLR4* expression levels close to or lower than pre-vaccination were observed. While observing an early activation of innate immune cell-related gene expression signatures, Fuller et al. noted a group of genes that were down-regulated through day 2 including genes related to pro-inflammatory cytokine response. Furthermore, *ICOS* and *ICAM1* genes were noted as down-regulated for most time points. Both genes were significantly down-regulated in our study at days 7 and 14. Despite these similarities, pronounced differences were overserved in that the increasing suppression of key innate response signaling pathways such as NF-κB and NOD-like receptor signaling, and the suppression of key inflammatory cytokines such as Il-1α, Il-1β, and TNF as well as *TLR4* was seen in our study only. Second, Fuller et al. reported a higher proportion of up-regulated than down-regulated genes relative to pre-vaccination reporting peak numbers at day 2, with 3–4-fold fewer genes reported at days 7 and 14. In contrast, we found an increase between days 1 and 14 in the magnitude of fold changes and the numbers of DE genes, with most genes being down-regulated. While one of the patterns identified by Fuller et al. (Pattern 8) showed a pronounced down-regulation of a group of genes starting at day 2 through day 14, an increase in suppression was not noted. We hypothesize that in addition to the mentioned differences in the assay, experimental design, sample size, and statistical analysis, the vaccine lot may have played an important role. We have shown previously that the new lot (DVC-LVS) of the older stockpiled USAMRIID-LVS vaccine induced significantly higher tularemia-specific microagglutination titers earlier in the time course [1]. One explanation for the observed differences between vaccine lots is that the older, stockpiled vaccine could have lost some of its viability. Alternatively, the application of modern CGMP methods (fermenter vs. shaker flasks), and difference in lineage or other vaccine manufacturing processes, could explain the observed discrepancies. As stronger immunosuppression was observed in our study, the former might be more plausible.

At the time of writing, the results from an Ebola metabolomics study were not available for comparison [12]. The metabolomics data from the herpes zoster vaccine [11] indicated that the greatest number of metabolites changed day 1 after vaccination (429 out 4048 features were significant using FDR < 0.1, negative ionization), but the study did not measure day 2 samples. Besides the larger number of differential metabolite features, the herpes zoster vaccine study also reported a larger number of metabolic pathways that were induced by vaccination. TCA cycle and nucleotide metabolism were reported for both studies, but the herpes zoster vaccine study identified additional pathways, such as glycosphingolipid metabolism and phosphatidylinositol phosphate metabolism. The difference between the two studies is likely a reflection of the difference in vaccines and their formulations. While the herpes zoster vaccine is a live-attenuated virus with carryover lipids from the manufacturing process in the formula, the DVC-LVS vaccine is based on a live-attenuated bacterium. In addition, vaccination routes were different.

## 5. Conclusions

Our transcriptomics results revealed that while some innate immune signaling pathways were activated, NF-κB, a key innate immune response pathway, and resulting key inflammatory cytokines were suppressed, implying that this *F. tularensis* live-attenuated vaccine altered human immune response. Nevertheless, humoral immune response signals were observed at day 7. The importance of metabolites in immunometabolism and immune signaling is being recognized in the field [70]. Our findings here, that metabolites were differentially abundant at early time points, were indicative of changes in glycolysis and TCA and predictive of adaptive immune responses. These findings suggest that further development in immunometabolomics will be beneficial for understanding the mechanisms of vaccines, and thus facilitate future vaccine development. Ultimately, well-designed systems biology studies that assess both host and pathogen responses simultaneously on the gene, protein, and metabolite level will be needed to shed light on the intricate interplay between the immune system and pathogen evasion mechanisms.

## Supplementary Materials

The following are available online at https://www.mdpi.com/2076-393X/8/3/412/s1, Supplemental results text, methods, figures, tables, and data: Data File S1: RNA-Seq read counts. Data File S2: Targeted metabolomics results as measured in log_2_ micromolar concentration. Data File S3: Untargeted metabolomics results (HILIC column) as measured in log_2_ peak intensity. Data File S4: Untargeted metabolomics results (C18 column) as measured in log_2_ peak intensity. Supplemental Text: Supplementary Text Manuscript Appendix for “Transcriptomic and metabolic responses to a live-attenuated Francisella tularensis vaccine” Data Table S1: Differentially expressed genes. Data Table S2: Pathways enriched in differentially expressed genes. Data Table S3: Differentially abundant metabolite features. Data Table S4: Human metabolic pathways enriched in differentially abundant metabolite features. Data Table S5: Metabolite features that best predicted peak microagglutination titer or CD4+ and CD8+ T cell activation. Data Table S6: Significantly correlated Blood Transcription Modules and metabolite clusters.
