# Supplementary material for: Transcriptomic and Metabolic Responses to a Live-Attenuated Francisella tularensis Vaccine
Source: Vaccines (Basel). 2020 Jul 24;8(3):412. doi: 10.3390/vaccines8030412 (PMC7563297; doi:10.3390/vaccines8030412)
Supplement: Supplementary file 1 [file vaccines-08-00412-s001.zip › fig/figure-1.pdf]

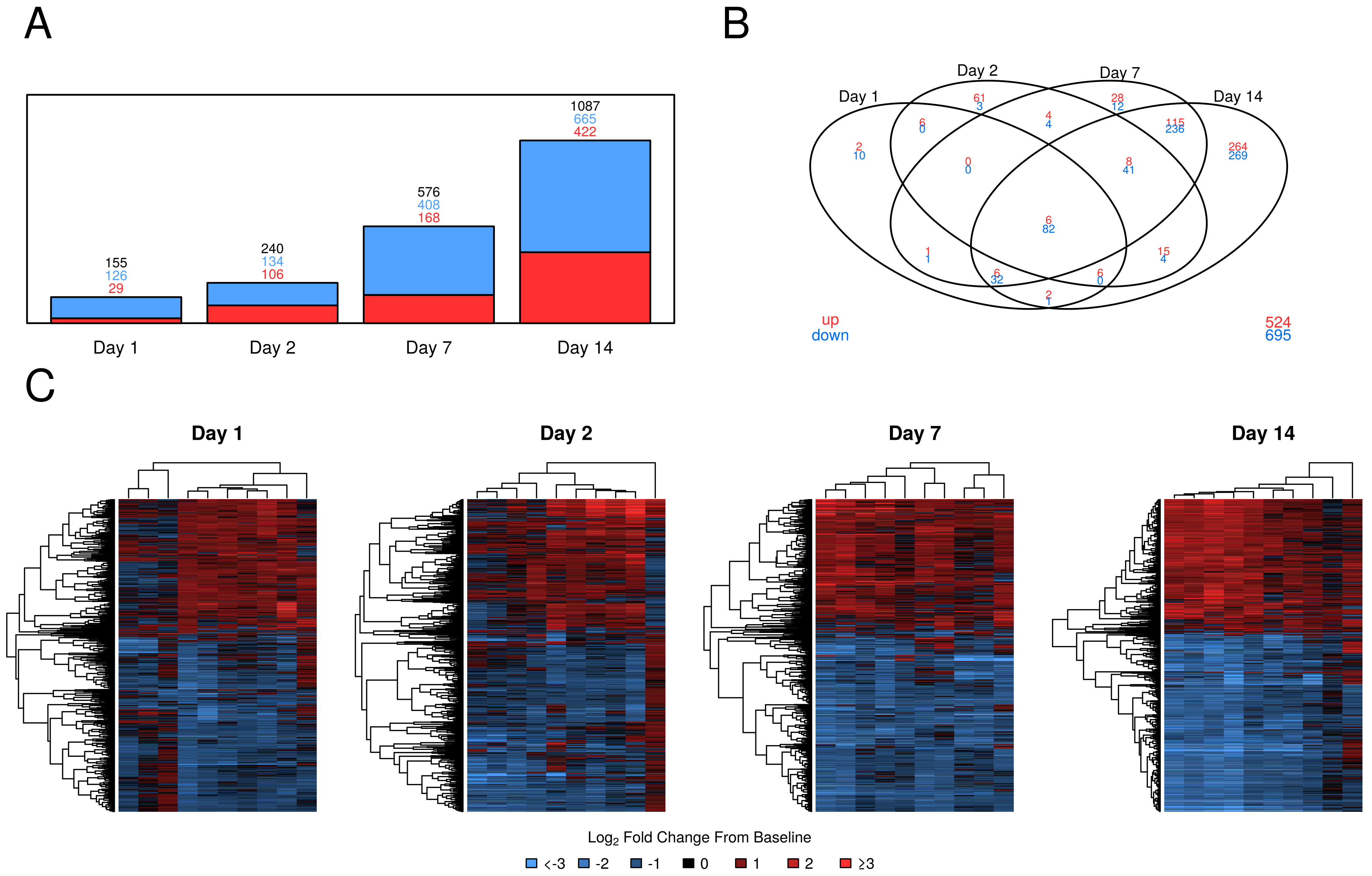
A
155
126
29
240
134
106
576
408
168
1087
665
422
Day 1
Day 2
Day 7
Day 14
B
Day 1
Day 2
Day 7
Day 14
2
10
6
0
61
3
4
4
28
12
115
236
264
269
0
0
8
41
6
82
1
1
15
4
6
32
6
0
2
1
up
down
524
695
C
Day 1
Day 2
Day 7
Day 14
Log2 Fold Change From Baseline
<-3
-2
-1
0
1
2
≥3
