# Supplementary material for: Transcriptomic and Metabolic Responses to a Live-Attenuated Francisella tularensis Vaccine
Source: Vaccines (Basel). 2020 Jul 24;8(3):412. doi: 10.3390/vaccines8030412 (PMC7563297; doi:10.3390/vaccines8030412)
Supplement: Supplementary file 1 [file vaccines-08-00412-s001.zip › fig/figure-2.pdf]

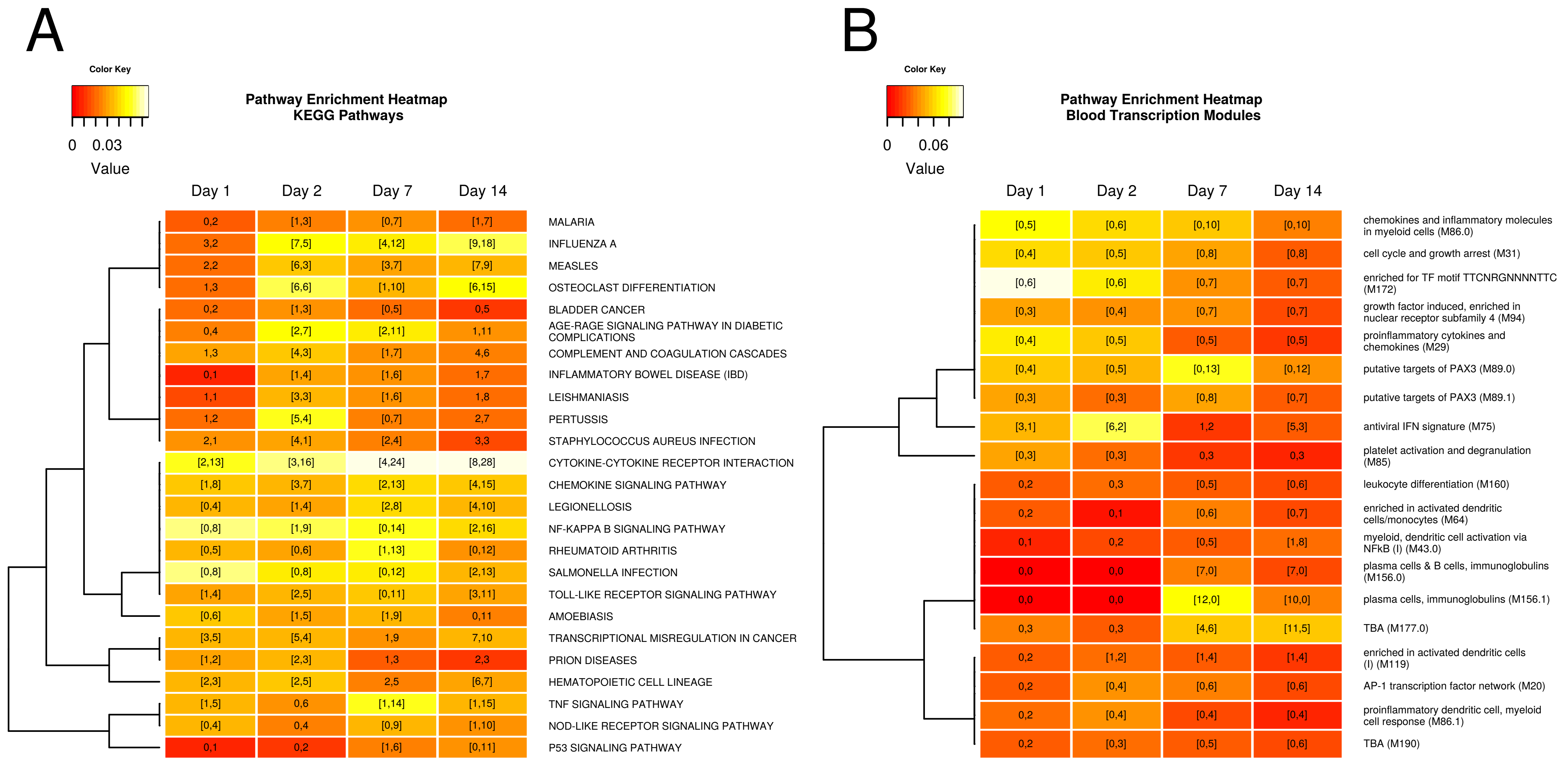
A
Color Key
0 0.03
Value
Pathway Enrichment Heatmap
KEGG Pathways
Day 1 Day 2 Day 7 Day 14
0,2 [1,3] [0,7] [1,7] MALARIA
3,2 [7,5] [4,12] [9,18] INFLUENZA A
2,2 [6,3] [3,7] [7,9] MEASLES
1,3 [6,6] [1,10] [6,15] OSTEOCLAST DIFFERENTIATION
0,2 [1,3] [0,5] 0,5 BLADDER CANCER
0,4 [2,7] [2,11] 1,11 AGE-RAGE SIGNALING PATHWAY IN DIABETIC COMPLICATIONS
1,3 [4,3] [1,7] 4,6 COMPLEMENT AND COAGULATION CASCADES
0,1 [1,4] [1,6] 1,7 INFLAMMATORY BOWEL DISEASE (IBD)
1,1 [3,3] [1,6] 1,8 LEISHMANIASIS
1,2 [5,4] [0,7] 2,7 PERTUSSIS
2,1 [4,1] [2,4] 3,3 STAPHYLOCOCCUS AUREUS INFECTION
[2,13] [3,16] [4,24] [8,28] CYTOKINE-CYTOKINE RECEPTOR INTERACTION
[1,8] [3,7] [2,13] [4,15] CHEMOKINE SIGNALING PATHWAY
[0,4] [1,4] [2,8] [4,10] LEGIONELLOSIS
[0,8] [1,9] [0,14] [2,16] NF-KAPPA B SIGNALING PATHWAY
[0,5] [0,6] [1,13] [0,12] RHEUMATOID ARTHRITIS
[0,8] [0,8] [0,12] [2,13] SALMONELLA INFECTION
[1,4] [2,5] [0,11] [3,11] TOLL-LIKE RECEPTOR SIGNALING PATHWAY
[0,6] [1,5] [1,9] 0,11 AMOEBIASIS
[3,5] [5,4] 1,9 7,10 TRANSCRIPTIONAL MISREGULATION IN CANCER
[1,2] [2,3] 1,3 2,3 PRION DISEASES
[2,3] [2,5] 2,5 [6,7] HEMATOPOIETIC CELL LINEAGE
[1,5] 0,6 [1,14] [1,15] TNF SIGNALING PATHWAY
[0,4] 0,4 [0,9] [1,10] NOD-LIKE RECEPTOR SIGNALING PATHWAY
0,1 0,2 [1,6] [0,11] P53 SIGNALING PATHWAY
B
Color Key
0 0.06
Value
Pathway Enrichment Heatmap
Blood Transcription Modules
Day 1 Day 2 Day 7 Day 14
[0,5] [0,6] [0,10] [0,10] chemokines and inflammatory molecules in myeloid cells (M86.0)
[0,4] [0,5] [0,8] [0,8] cell cycle and growth arrest (M31)
[0,6] [0,6] [0,7] [0,7] enriched for TF motif TTCNRGNNNNTTC (M172)
[0,3] [0,4] [0,7] [0,7] growth factor induced, enriched in nuclear receptor subfamily 4 (M94)
[0,4] [0,5] [0,5] [0,5] proinflammatory cytokines and chemokines (M29)
[0,4] [0,5] [0,13] [0,12] putative targets of PAX3 (M89.0)
[0,3] [0,3] [0,8] [0,7] putative targets of PAX3 (M89.1)
[3,1] [6,2] 1,2 [5,3] antiviral IFN signature (M75)
[0,3] [0,3] 0,3 0,3 platelet activation and degranulation (M85)
0,2 0,3 [0,5] [0,6] leukocyte differentiation (M160)
0,2 0,1 [0,6] [0,7] enriched in activated dendritic cells/monocytes (M64)
0,1 0,2 [0,5] [1,8] myeloid, dendritic cell activation via NFkB (I) (M43.0)
0,0 0,0 [7,0] [7,0] plasma cells & B cells, immunoglobulins (M156.0)
0,0 0,0 [12,0] [10,0] plasma cells, immunoglobulins (M156.1)
0,3 0,3 [4,6] [11,5] TBA (M177.0)
0,2 [1,2] [1,4] [1,4] enriched in activated dendritic cells (I) (M119)
0,2 [0,4] [0,6] [0,6] AP-1 transcription factor network (M20)
0,2 [0,4] [0,4] [0,4] proinflammatory dendritic cell, myeloid cell response (M86.1)
0,2 [0,3] [0,5] [0,6] TBA (M190)
