# Supplementary material for: Transcriptomic and Metabolic Responses to a Live-Attenuated Francisella tularensis Vaccine
Source: Vaccines (Basel). 2020 Jul 24;8(3):412. doi: 10.3390/vaccines8030412 (PMC7563297; doi:10.3390/vaccines8030412)
Supplement: Supplementary file 1 [file vaccines-08-00412-s001.zip › fig/figure-4.pdf]

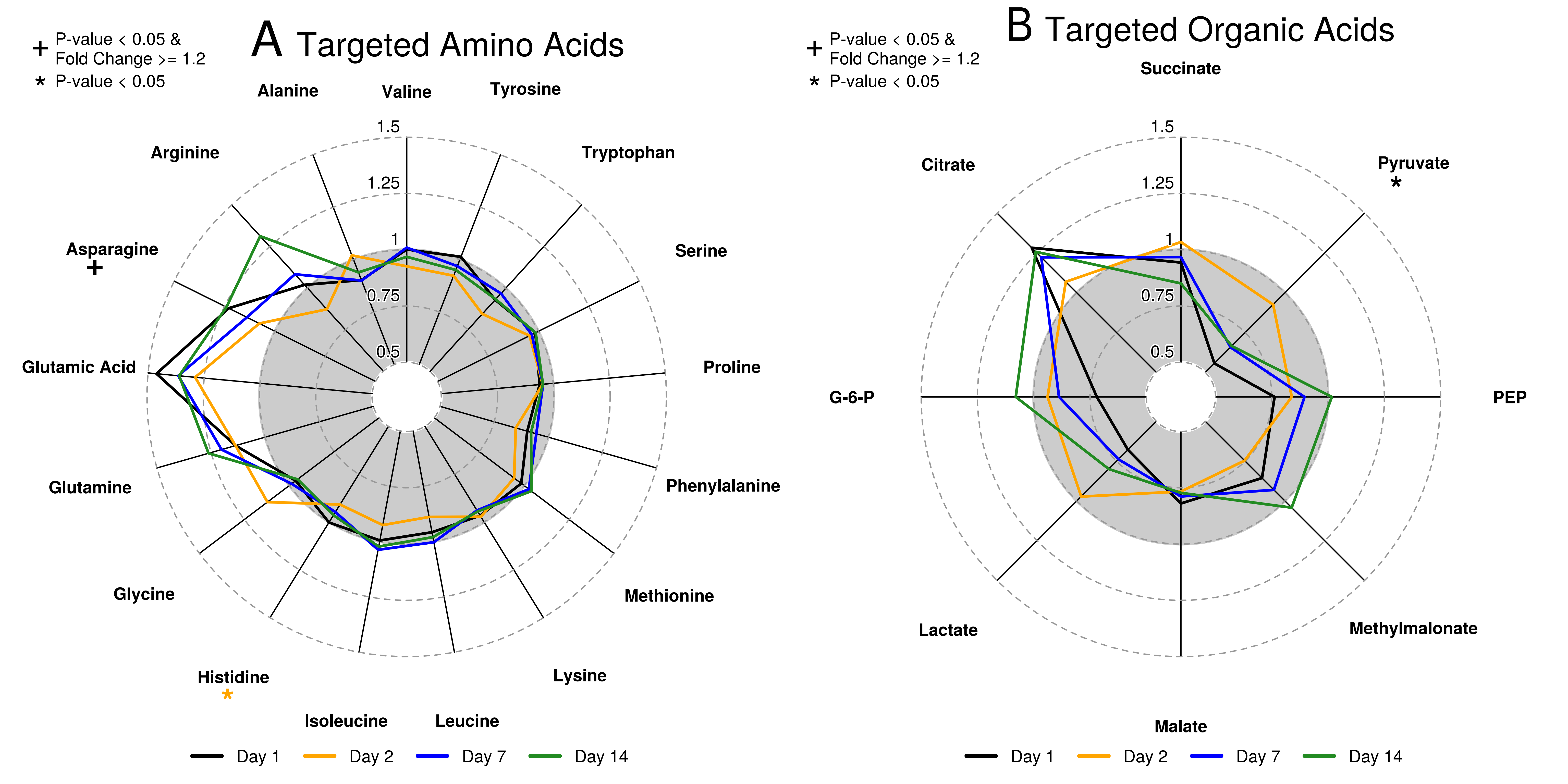

A Targeted Amino Acids
+ P-value < 0.05 & Fold Change >= 1.2
* P-value < 0.05
Alanine
Valine
Tyrosine
Arginine
Tryptophan
Asparagine +
Serine
Glutamic Acid
Proline
Glutamine
Phenylalanine
Glycine
Methionine
Histidine *
Lysine
Isoleucine
Leucine
1.5
1.25
1
0.75
0.5
Day 1
Day 2
Day 7
Day 14
B Targeted Organic Acids
+ P-value < 0.05 & Fold Change >= 1.2
* P-value < 0.05
Succinate
Citrate
Pyruvate *
G-6-P
PEP
Lactate
Methylmalonate
Malate
1.5
1.25
1
0.75
0.5
Day 1
Day 2
Day 7
Day 14
