# Supplementary material for: Transcriptomic and Metabolic Responses to a Live-Attenuated Francisella tularensis Vaccine
Source: Vaccines (Basel). 2020 Jul 24;8(3):412. doi: 10.3390/vaccines8030412 (PMC7563297; doi:10.3390/vaccines8030412)

Metabolite Baseline Response Heatmap
HILIC LC Column, Day 2

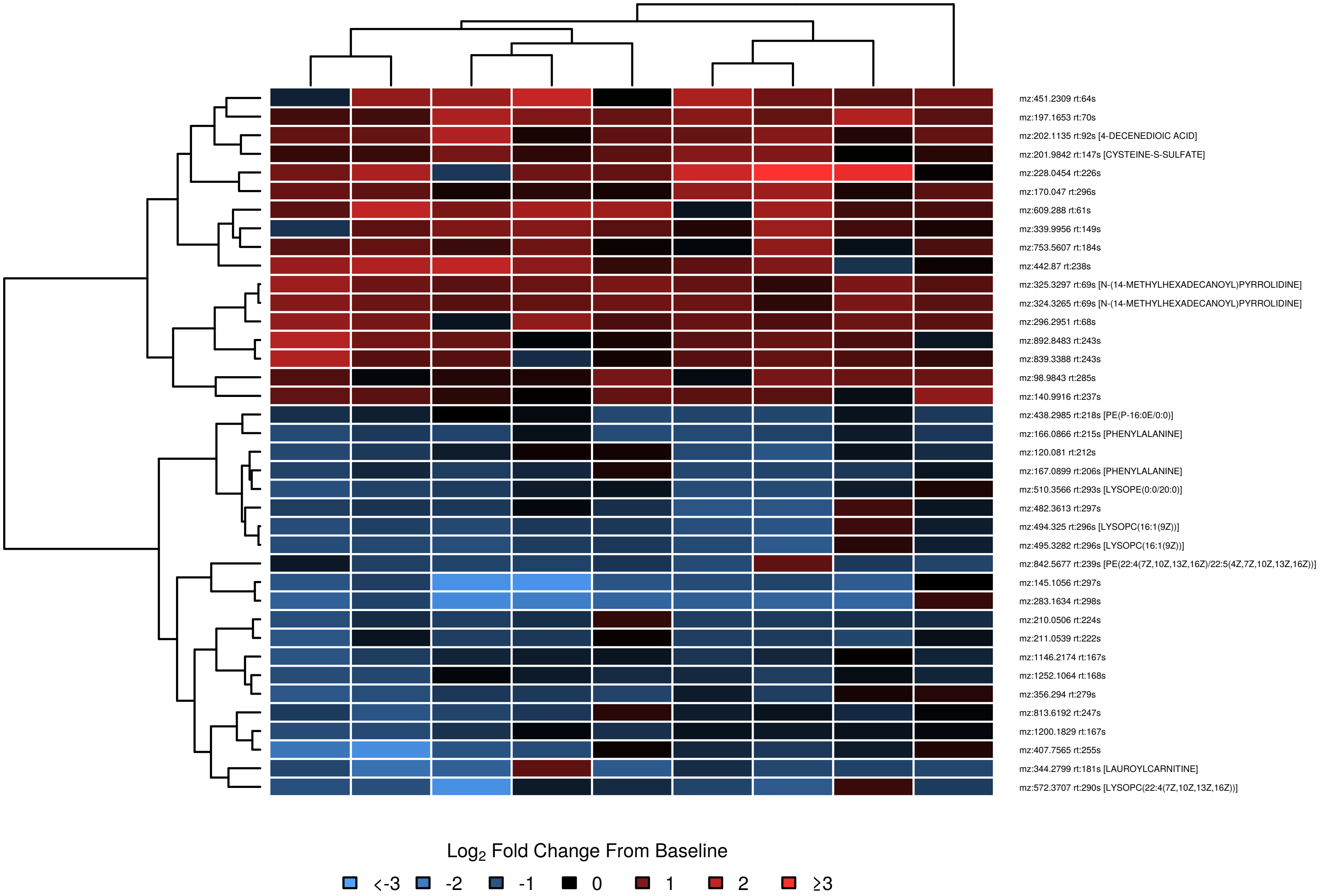

Supplement: Supplementary file 1 [file vaccines-08-00412-s001.zip › fig/figure-5.pdf]
