# Supplementary material for: Transcriptomic and Metabolic Responses to a Live-Attenuated Francisella tularensis Vaccine
Source: Vaccines (Basel). 2020 Jul 24;8(3):412. doi: 10.3390/vaccines8030412 (PMC7563297; doi:10.3390/vaccines8030412)
Supplement: Supplementary file 1 [file vaccines-08-00412-s001.zip › fig/figure-6.pdf]

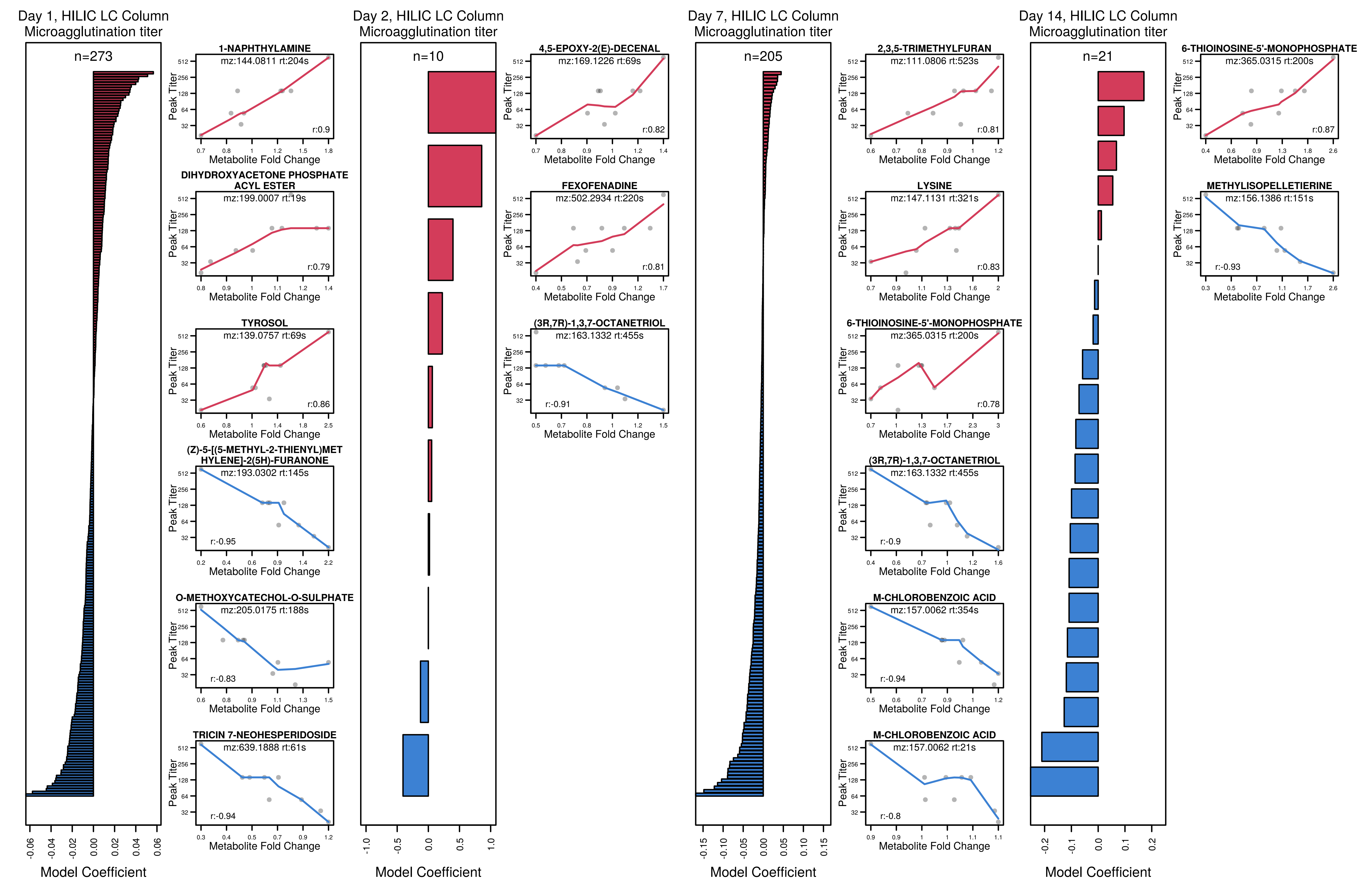

Day 1, HILIC LC Column
Microagglutination titer
n=273
Model Coefficient
1-NAPHTHYLAMINE
mz:144.0811 rt:204s
r:0.9
DIHYDROXYACETONE PHOSPHATE ACYL ESTER
mz:199.0007 rt:19s
r:0.79
TYROSOL
mz:139.0757 rt:69s
r:0.86
(Z)-5-[(5-METHYL-2-THIENYL)METHYLENE]-2(5H)-FURANONE
mz:193.0302 rt:145s
r:-0.95
O-METHOXYCATECHOL-O-SULPHATE
mz:205.0175 rt:188s
r:-0.83
TRICIN 7-NEOHESPERIDOSIDE
mz:639.1888 rt:61s
r:-0.94
Day 2, HILIC LC Column
Microagglutination titer
n=10
Model Coefficient
4,5-EPOXY-2(E)-DECENAL
mz:169.1226 rt:69s
r:0.82
FEXOFENADINE
mz:502.2934 rt:220s
r:0.81
(3R,7R)-1,3,7-OCTANETRIOL
mz:163.1332 rt:455s
r:-0.91
Day 7, HILIC LC Column
Microagglutination titer
n=205
Model Coefficient
2,3,5-TRIMETHYLFURAN
mz:111.0806 rt:523s
r:0.81
LYSINE
mz:147.1131 rt:321s
r:0.83
6-THIOINOSINE-5'-MONOPHOSPHATE
mz:365.0315 rt:200s
r:0.78
(3R,7R)-1,3,7-OCTANETRIOL
mz:163.1332 rt:455s
r:-0.9
M-CHLOROBENZOIC ACID
mz:157.0062 rt:354s
r:-0.94
M-CHLOROBENZOIC ACID
mz:157.0062 rt:21s
r:-0.8
Day 14, HILIC LC Column
Microagglutination titer
n=21
Model Coefficient
6-THIOINOSINE-5'-MONOPHOSPHATE
mz:365.0315 rt:200s
r:0.87
METHYLISOPELLETIERINE
mz:156.1386 rt:151s
r:-0.93
Peak Titer
Metabolite Fold Change
