# Supplementary figures and images for: Transcriptomic and Metabolic Responses to a Live-Attenuated Francisella tularensis Vaccine
Source: Vaccines (Basel). 2020 Jul 24;8(3):412. doi: 10.3390/vaccines8030412 (PMC7563297; doi:10.3390/vaccines8030412)

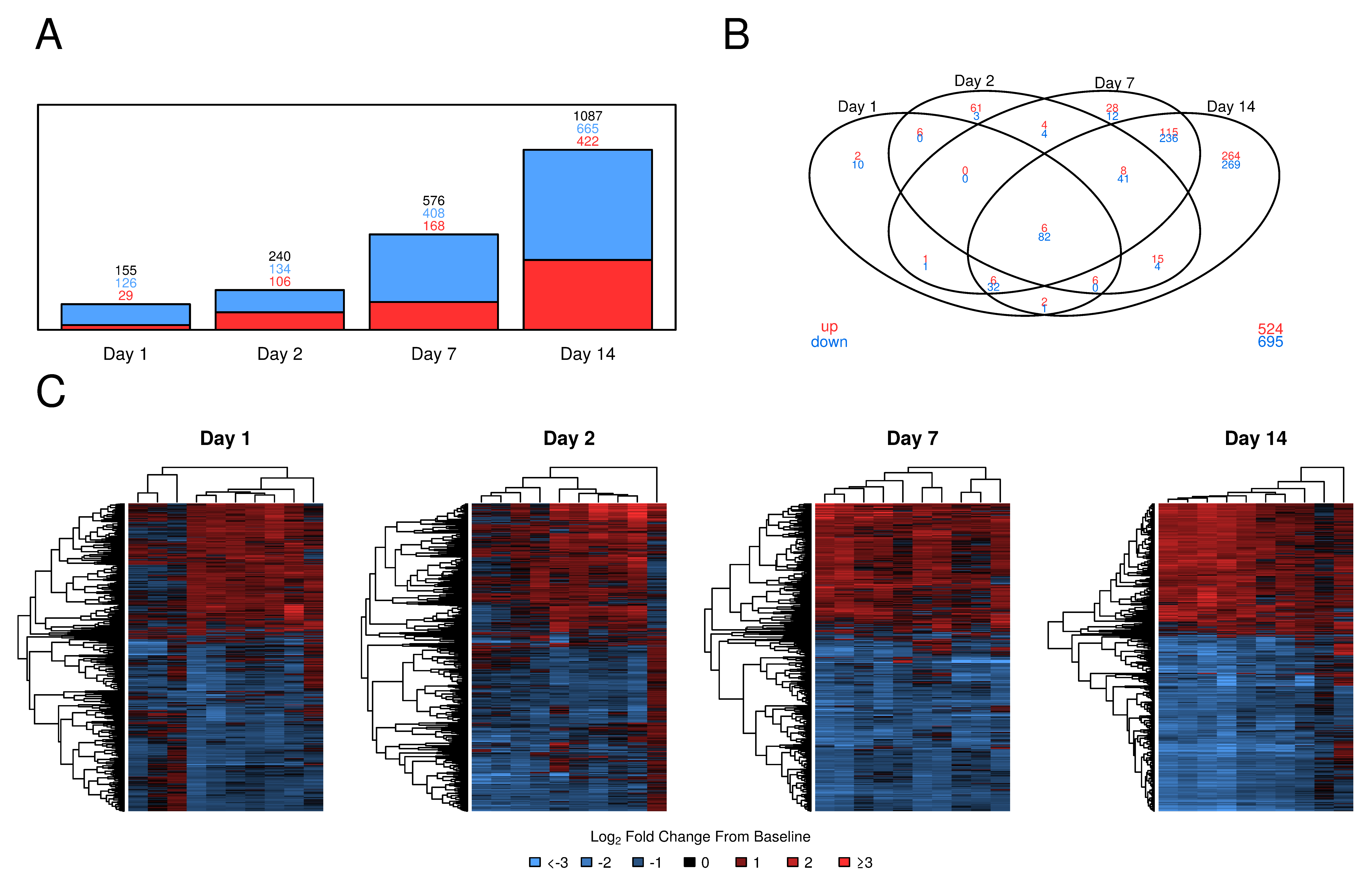

Supplement: Supplementary file 1 [file vaccines-08-00412-s001.zip › fig/figure-1.png]

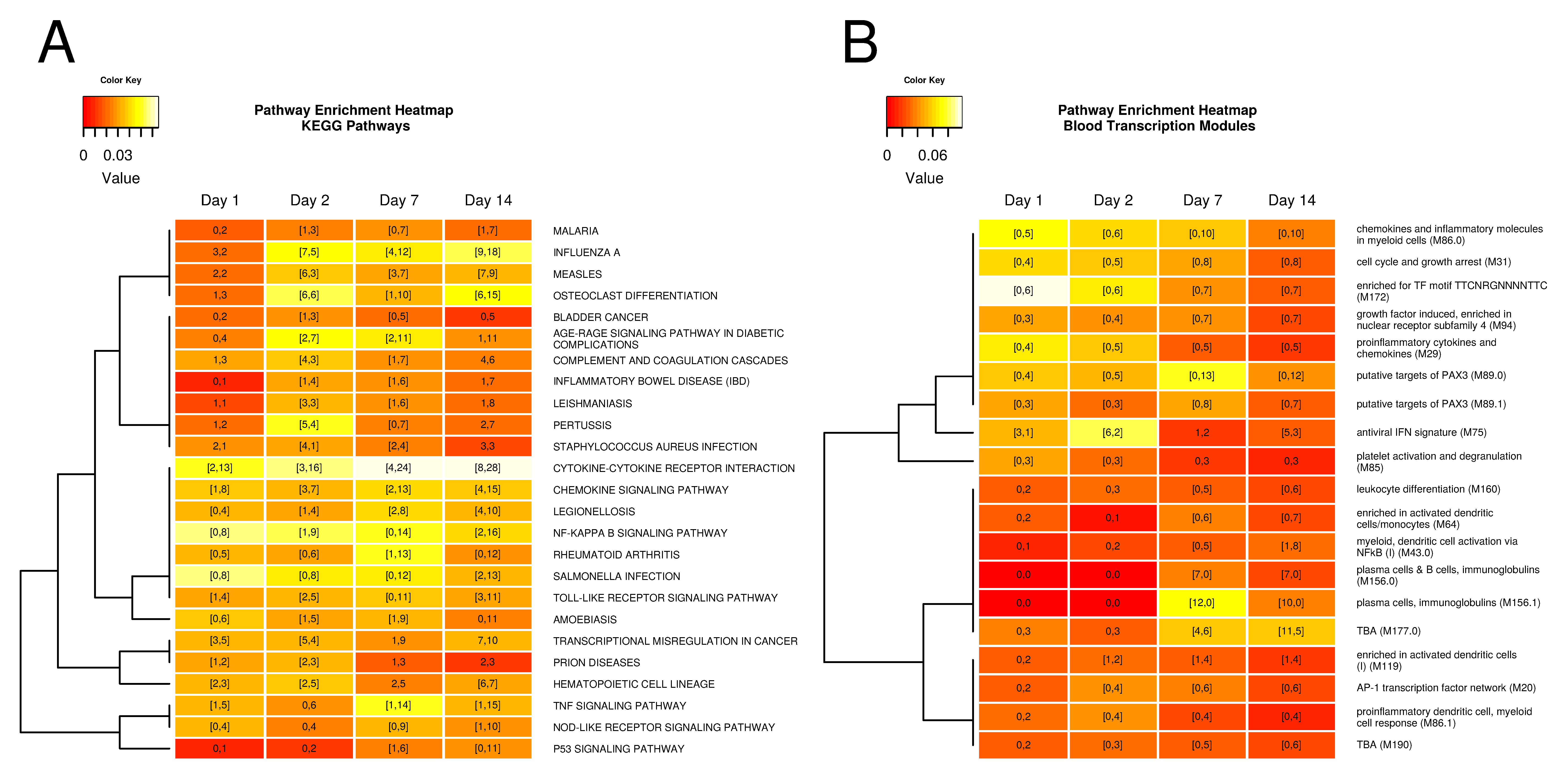

Supplement: Supplementary file 1 [file vaccines-08-00412-s001.zip › fig/figure-2.png]

A

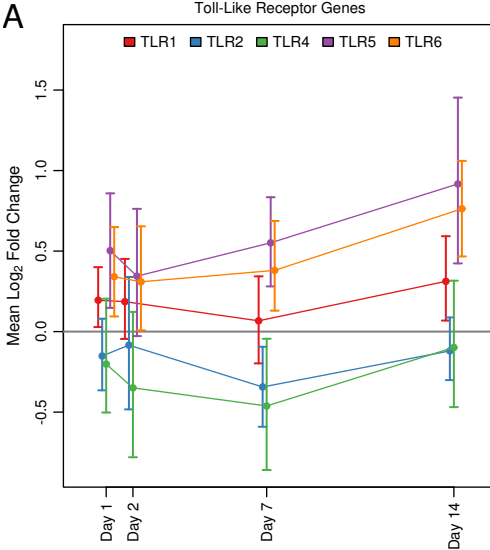

B

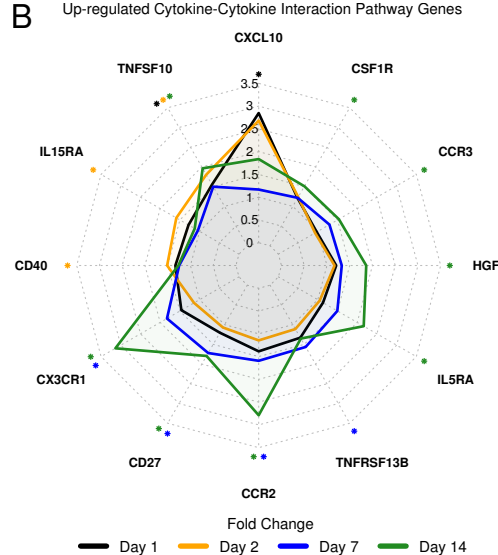

C

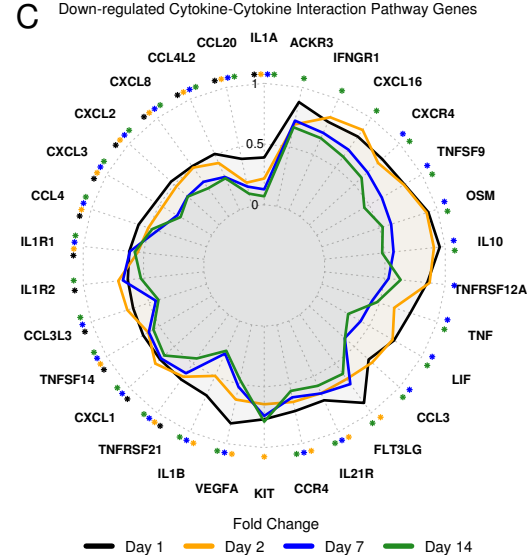

Supplement: Supplementary file 1 [file vaccines-08-00412-s001.zip › fig/figure-3.pdf]

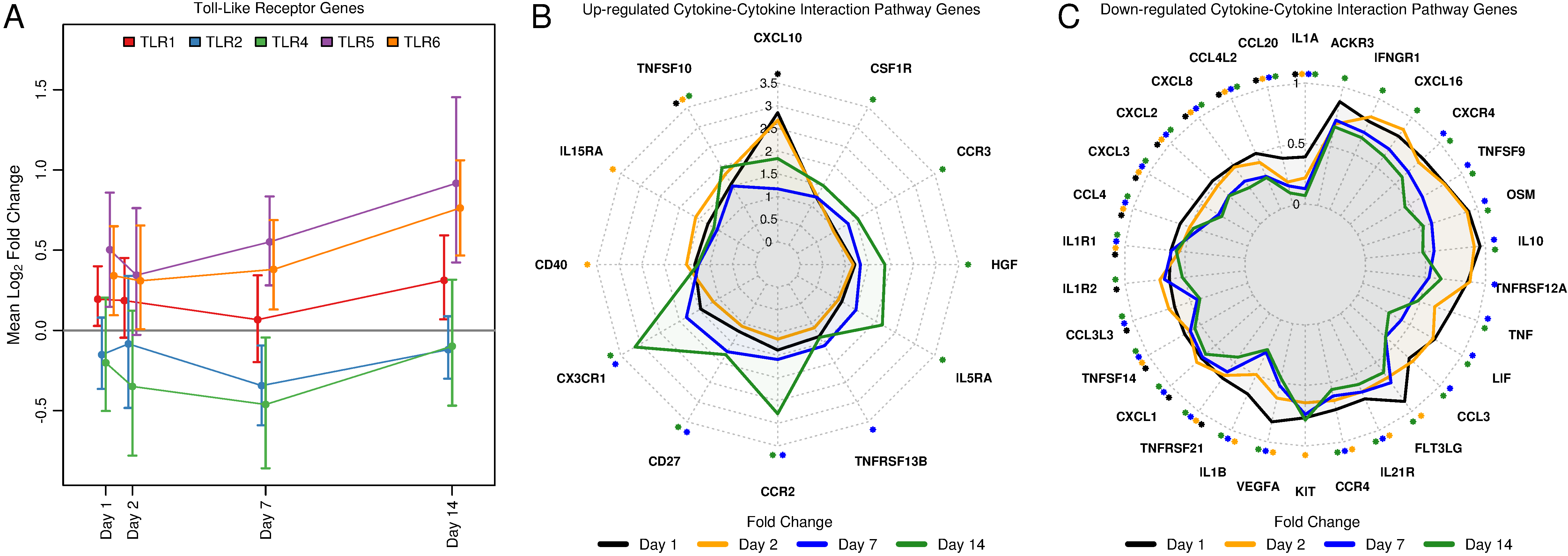

Supplement: Supplementary file 1 [file vaccines-08-00412-s001.zip › fig/figure-3.png]

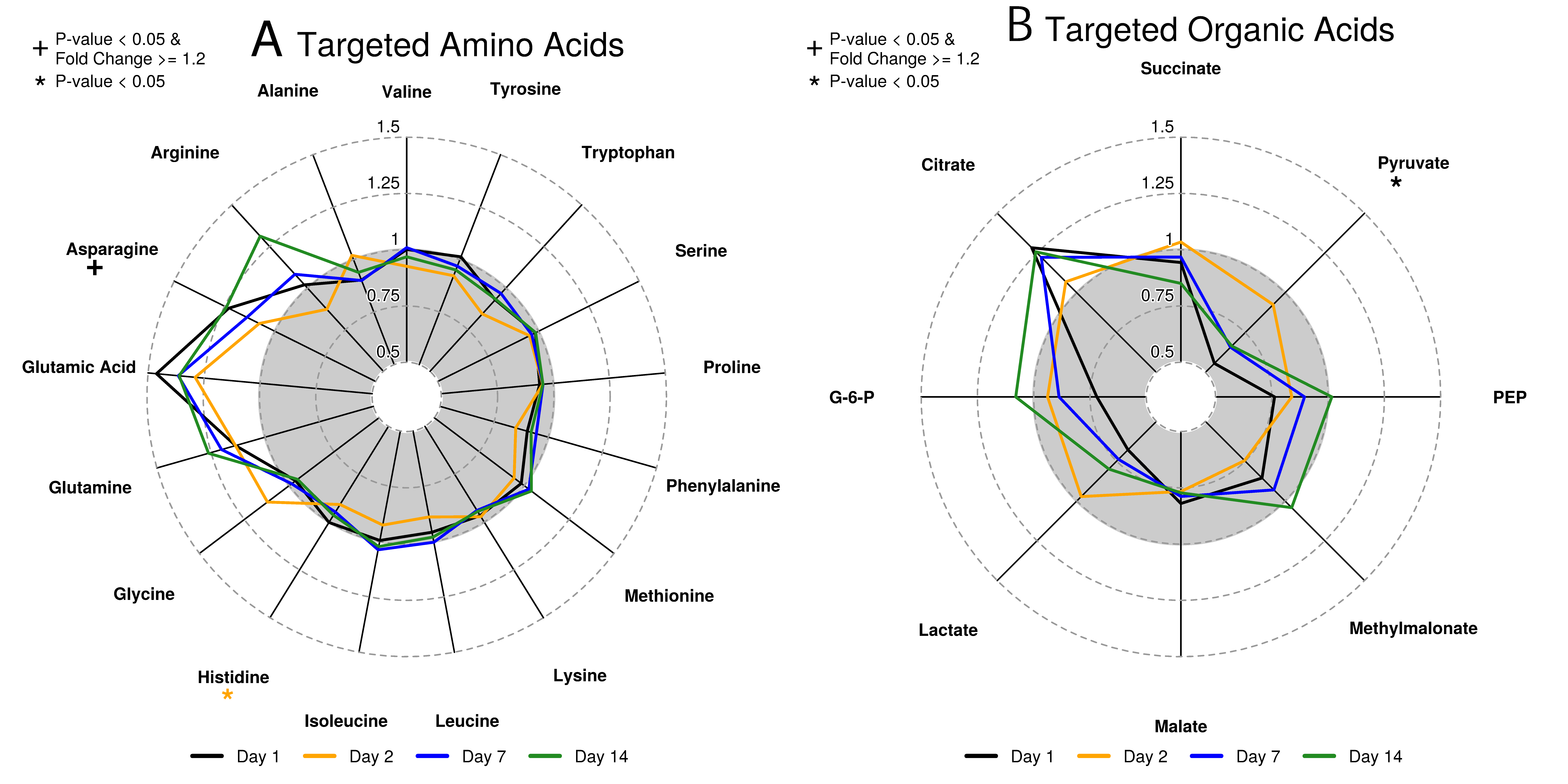

Supplement: Supplementary file 1 [file vaccines-08-00412-s001.zip › fig/figure-4.png]

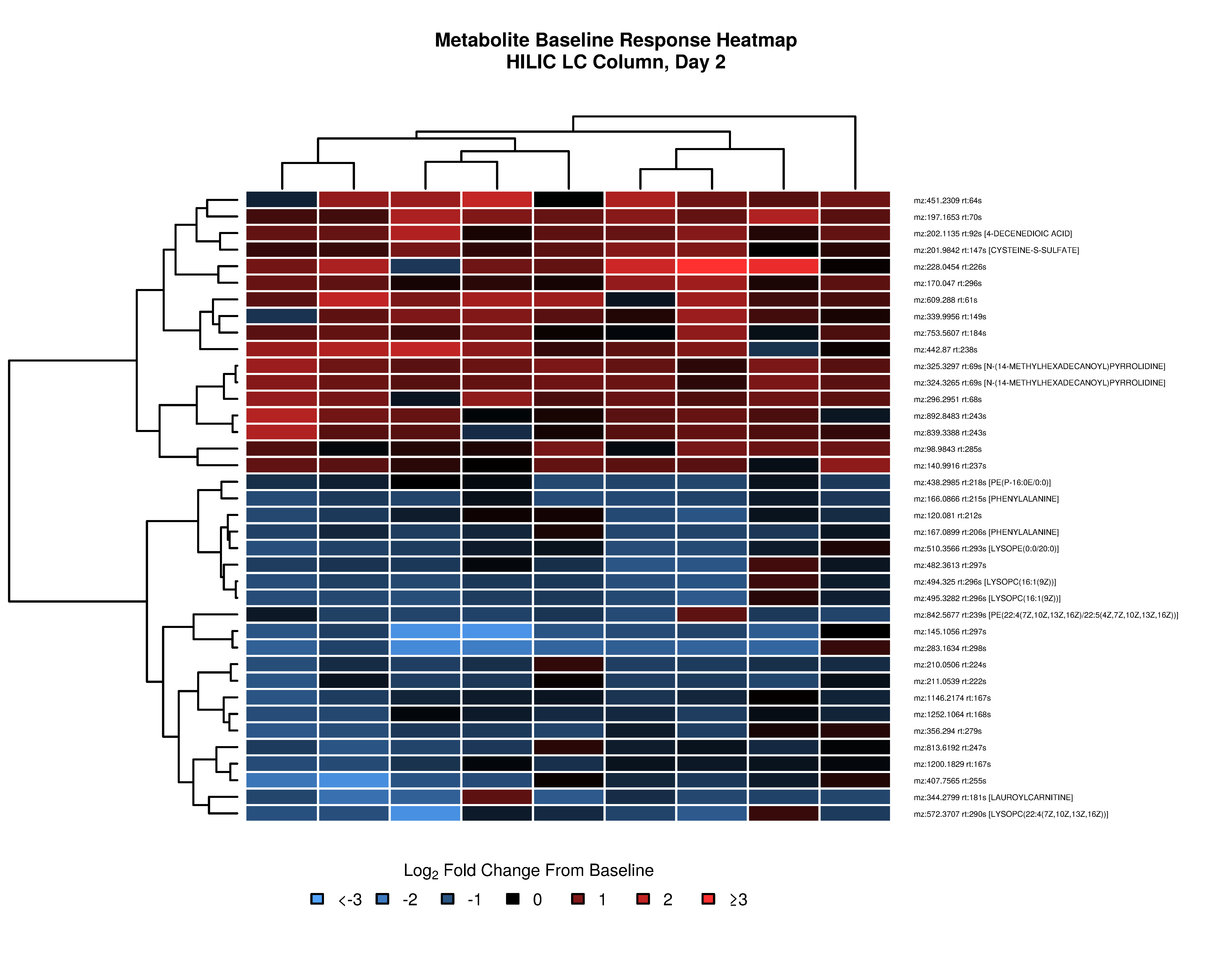

Supplement: Supplementary file 1 [file vaccines-08-00412-s001.zip › fig/figure-5.png]

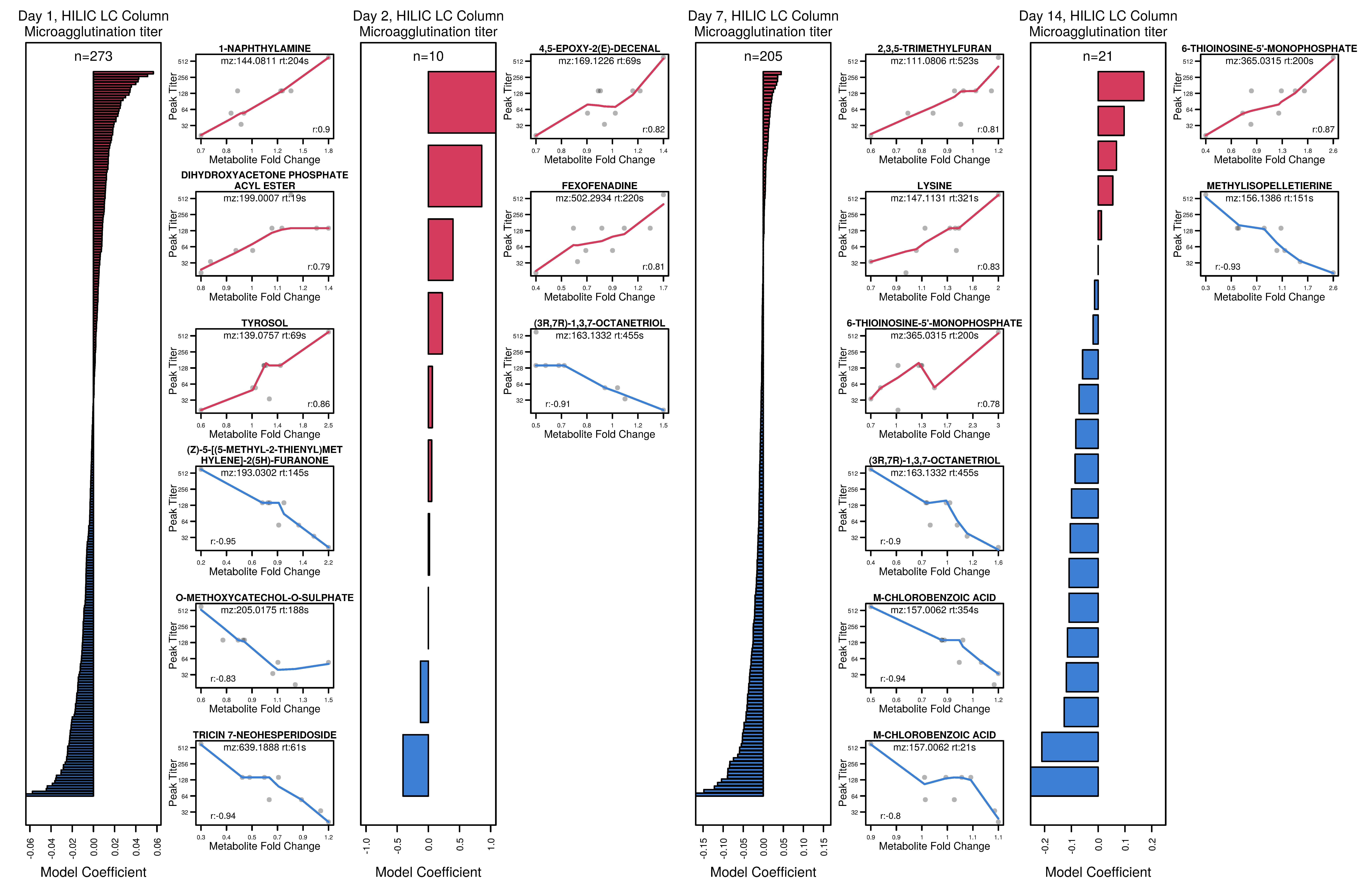

Supplement: Supplementary file 1 [file vaccines-08-00412-s001.zip › fig/figure-6.png]

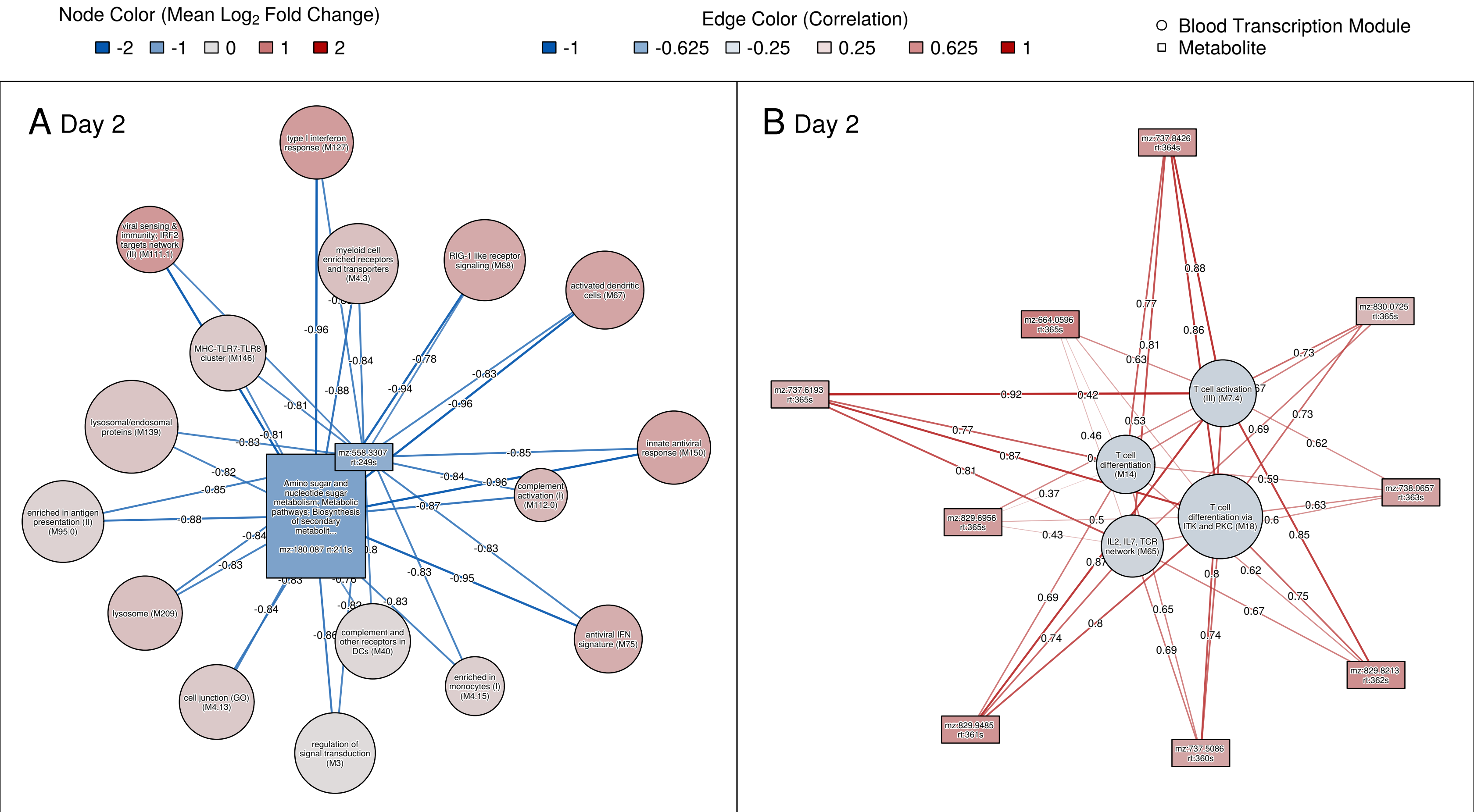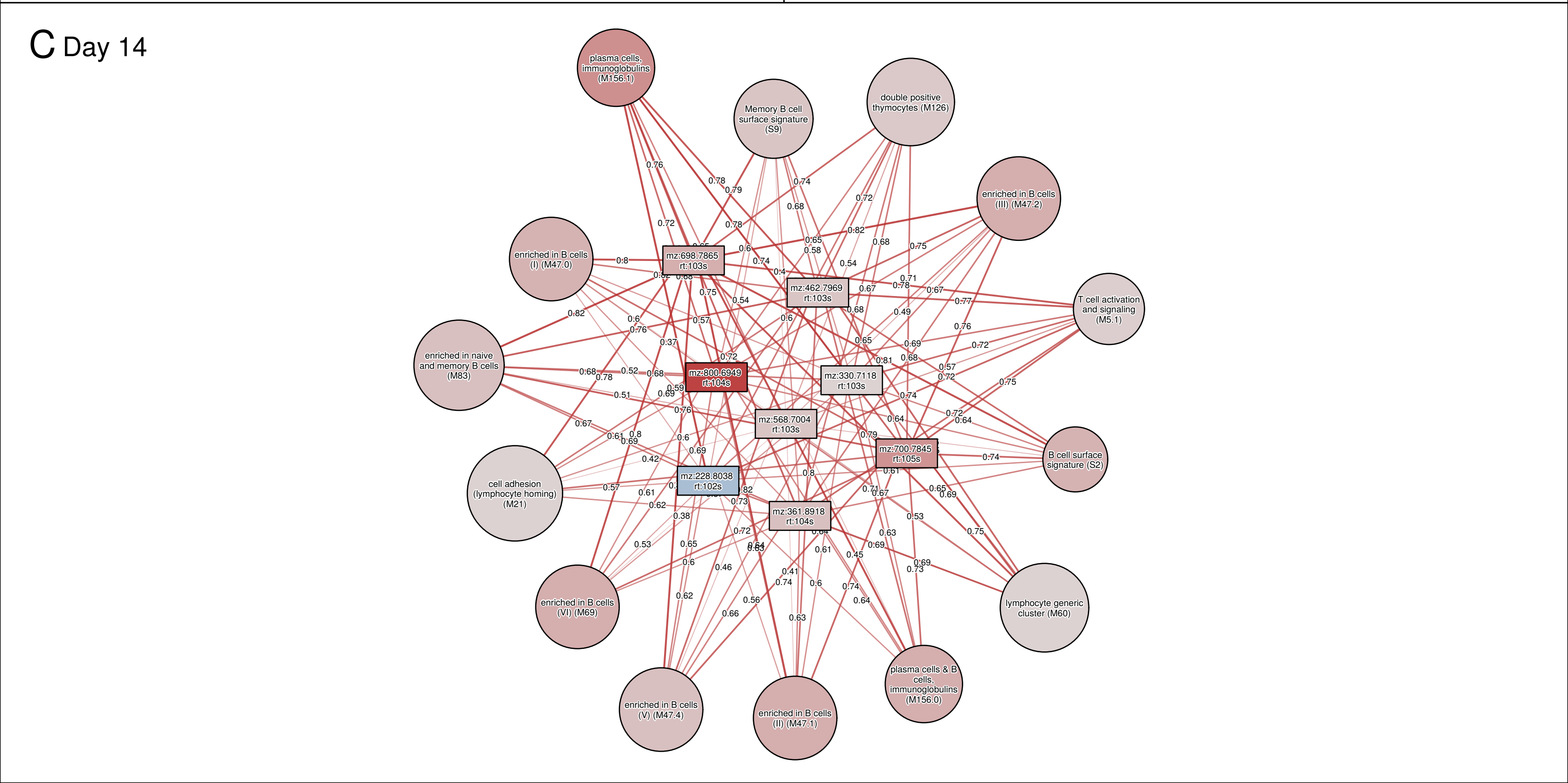

Supplement: Supplementary file 1 [file vaccines-08-00412-s001.zip › fig/figure-7.pdf]
